# Social Crowding and Shared Consumption with Close Friends Versus Strangers

**DOI:** 10.3390/bs16081394

**Published:** 2026-08-14

**Authors:** Lingling He, Wencai Zhou, Yuqi Qian, Shichang Liang, Jing Lin, Lingrui Tu

**Affiliations:** 1 School of Economics and Management, Nanning Normal University, No. 3, Hexing Road, Qingxiu District, Nanning 530299, China; helingling@nnnu.edu.cn (L.H.); wenzai981025@163.com (W.Z.); qk041709@126.com (Y.Q.); sopeninim@gmail.com (J.L.); 2 School of Business, Guangxi University, No. 100, Daxue Dong Road, Nanning 530004, China; 3 Norwich Business School, University of East Anglia, Norwich Research Park, Norwich NR4 7TJ, UK; lr2bizstu@gmail.com

**Keywords:** social crowding, shared consumption, psychological distance, resource mindset, construal level theory

## Abstract

This study investigates how social crowding (e.g., overcrowded environments) influences individuals’ preferences for different modes of shared consumption (sharing-in vs. sharing-out). While existing research has predominantly explored shared consumption through the perspective of non-psychosocial environmental factors (e.g., time scarcity), this study shifts the focus to psychosocial environmental factors by analyzing the underlying mechanism through which social crowding shapes preferences for these modes of shared consumption. We conducted one field experiment and three scenario-based laboratory experiments to manipulate social crowding (vs. non-social crowding) across three distinct contexts: (1) restaurant-based social scenarios, (2) shopping mall consumption contexts, and (3) beach tourism settings. Across these experiments, diverse categories of shared goods were employed to examine participants’ preferences for sharing-in versus sharing-out. The findings demonstrate that social crowding increases individuals’ preference for sharing with close friends (i.e., sharing-in), whereas non-social crowding enhances their preference for sharing with strangers (i.e., sharing-out). This relationship is mediated by psychological distance. Furthermore, resource mindset (scarcity vs. abundance) moderates this effect. Specifically, under a scarcity mindset, social crowding strengthens preferences for sharing-in, whereas under an abundance mindset, it promotes preferences for sharing-out. By conceptualizing shared consumption from a psychosocial environmental perspective, this research extends current understanding of how environmental social cues shape interpersonal consumption decisions and provides practical implications for collaborative consumption platforms, hospitality services, retail environments, tourism destinations, and public service design. Specifically, platform managers and service designers can strategically optimize spatial layouts and perceived crowding levels to strengthen social connectedness among close friends or foster positive interactions between strangers.

## 1. Introduction

In everyday life, shared consumption has become increasingly embedded in routine social interactions. Beyond specialized public health amenities (e.g., public hand sanitizers, sunscreen dispensers, and hotel toiletries), diverse sharing platforms encompassing accommodation, ride-sharing, co-working spaces, and parking services have increasingly permeated individuals’ daily lives (Khalek & Chakraborty, 2022; Khodayari et al., 2025). Market data underscore this trend: the public hand sanitizer market is projected to reach $182 million by 2027 (Allied Market Research, 2024), while over 7000 public sunscreen dispensers have been deployed across public locations in the United States (IMPACT Melanoma, 2023). Notably, many of these shared consumption opportunities occur within densely populated or highly crowded environments. Consider an everyday scenario: while dining in a restaurant, a single bottle of public hand sanitizer is placed on the table. You are seated alongside two distinct types of individuals, close friends on one hand, and unfamiliar strangers on the other. If both parties require the sanitizer simultaneously, with whom would you be more inclined to share? More importantly, would you change your preference if you were immersed in a bustling, crowded atmosphere versus an uncrowded one? This fundamental behavioral paradox lies at the heart of what the present research aims to resolve.

Shared consumption has permeated daily life, stimulating diverse individual consumer behaviors through various sharing modes (Ackermann & Tunn, 2024). Based on the social relationship with the sharing recipient, shared consumption can be broadly classified into sharing-in (i.e., sharing with close friends) and sharing-out (i.e., sharing with strangers) (Boothby et al., 2016). As shared consumption increasingly integrates into society, researchers have turned their attention to its underlying behavioral patterns, expanding the literature on preferences for different sharing modes. For instance, under conditions of limited time resources, individuals exhibit a stronger preference for sharing unique and memorable experiences with close companions to maintain and deepen their relationships (Garcia-Rada & Kim, 2021). Similarly, having physical contact during shared consumption yields higher satisfaction when sharing with friends, whereas no such change is observed when sharing with strangers (Enestrom et al., 2023). In interpersonal domains, supportive sibling relationships within the same university can strengthen emotional ties, rendering individuals more willing to share daily experiences (Younkin et al., 2021). Furthermore, individual traits play a significant role; individuals with attachment anxiety are more inclined to share with close companions, particularly when disclosing negative life experiences (Snyder et al., 2024), as cultivating intimacy inherently fosters a greater depth of interpersonal sharing (Bedrov & Gable, 2024). Despite these valuable insights, empirical evidence regarding the specific antecedents that shape individuals’ preferences for these distinct shared consumption modes remains scant. Crucially, the vast majority of prior research has adopted the perspective of non-psychosocial environmental factors, focusing strictly on variables such as time scarcity, physical proximity, sibling relationship, or attachment anxiety. The potential impact of psychosocial environmental factors remains heavily under-explored. To address this research gap, the present study investigates how social crowding (vs. non-social crowding) influences individuals’ preferences for different shared consumption modes (sharing-in vs. sharing-out). Prior research suggests that social crowding in bustling retail spaces tends to increase interpersonal interaction and reinforce connections with close others (Consiglio et al., 2018). In contrast, non-social crowding may promote openness and exploratory interactions with strangers (Hou et al., 2021). Accordingly, individuals in social crowding may be more inclined toward sharing-in, while those in non-social crowding may be more likely to engage in sharing-out. We further argue that this effect is mediated by psychological distance, as social crowding reduces perceived distance and enhances feelings of belonging and safety (Liu et al., 2020). In contrast, non-social crowding may increase perceived distance and encourage social exploration (So et al., 2019). While Social Identity Theory and Attachment Theory provide valuable insights into group-based categorization and stable relational security, respectively, Construal Level Theory (CLT) offers a more precise theoretical framework for our proposed mechanism. Specifically, CLT explains how social crowding alters an individual’s subjective psychological distance, which in turn systematically redirects their sharing-target preferences. By positioning psychological distance as the mediating mechanism, CLT directly addresses the process through which environmental psychosocial factors reorganize consumer relational orientations in real-time. Grounded in construal level theory (Trope et al., 2007), we posit that this effect is mediated by psychological distance.

Finally, we propose that resource mindset moderates these effects. Under conditions of perceived scarcity, individuals prioritize security and affiliation, making them more responsive to the affiliative cues present in social crowding and thus more likely to engage in sharing-in (Mehta & Zhu, 2016). In contrast, individuals with an abundance mindset tend to emphasize variety and exploration, which may increase their willingness to engage in sharing-out, even within social crowding, such as exchanging resources in busy marketplaces or interacting with unfamiliar others in dense public settings (Hebrok & Boks, 2017; Neville et al., 2022).

This study makes three theoretical contributions to the existing literature. First, by introducing a perspective of psychosocial environmental factors into the realm of shared consumption, this study effectively bridges a notable gap in the literature. Prior research has predominantly focused on factors such as time scarcity and physical proximity (Garcia-Rada & Kim, 2021; Enestrom et al., 2023). Second, to the best of our knowledge, this study is among the first to establish a theoretical link between social crowding and psychological distance within the dimension of crowding density. Crucially, it provides empirical evidence that social crowding versus non-social crowding exerts distinct, divergent effects on individuals’ psychological distance. Third, this research elucidates the moderating role of resource mindset (scarcity vs. abundance) on the relationship between social crowding and shared consumption preferences. It demonstrates that consumers’ preferences for sharing modes dynamically shift with environmental crowding, exhibiting differentiated behavioral characteristics depending on their underlying resource mindset. Beyond its theoretical advancements, this research offers concrete, actionable insights for practitioners across collaborative platforms, retail, and service environments. First, managers of sharing platforms and physical service spaces (e.g., restaurants, co-working spaces, and public facilities) can strategically utilize spatial design and crowding density to foster desired customer behaviors, promoting inner-sharing initiatives in crowded settings and encouraging outer-sharing in spacious ones. Second, marketers can tailor promotional framing by aligning consumers’ resource mindsets (scarcity vs. abundance framing) with environmental density to optimize user engagement and platform adoption.

## 2. Theoretical Background

### 2.1. Social Crowding

Social crowding refers to the psychological experience that arises when individuals are exposed to densely populated social environments, such as subway cars during rush hour or retail stores during peak sales periods, which exemplify settings in which personal space is substantially constrained (Huang et al., 2018). In contrast, non-social crowding refers to the psychological experience that arises when individuals are situated in sparsely populated environments, such as an uncrowded subway car or a nearly empty retail store, where the lack of social presence may trigger compensatory behavioral responses (Maeng et al., 2013). Prior research distinguishes between two forms of crowding: social crowding, which reflects the number of people within a given space, and spatial crowding, which captures the subjective perception of insufficient physical space even in the absence of high density (Y. Cheng et al., 2024). With increasing global population density, social crowding has become a pervasive feature of modern life, attracting growing scholarly attention (Z. Sun et al., 2026). The present research examines how social crowding shapes interpersonal dynamics, particularly within the relational context of shared consumption. We summarize the existing literature on social crowding (see Table A1 in Appendix A).

Extant research on social crowding presents a complex, often contradictory landscape regarding its behavioral consequences, oscillating between theories of social withdrawal and social facilitation. From one perspective, crowding is consistently linked to negative outcomes. Physiologically, high social density can induce discomfort, elevate body temperature, and heighten disease transmission risks (Herath et al., 2024). Psychologically, close interpersonal proximity often triggers amygdala-based stress responses (Kennedy et al., 2009), contributing to symptoms such as dizziness, chest tightness, and fatigue (Z. Zhang et al., 2023). Furthermore, social crowding frequently diminishes individuals’ perceived control, social status, and sense of belonging (O’Guinn et al., 2015; Huang et al., 2018), which may evoke hostility, frustration, and a reduced willingness to share information (Wang & Zhang, 2025).

Conversely, a competing body of research suggests that crowding can paradoxically foster social cohesion. Space constraints have been shown to heighten motivations for autonomy and self-expression (Wicklund, 1974). Social crowding has been found to facilitate interpersonal communication (Consiglio et al., 2018), increase preferences for socially oriented products (Peng et al., 2024), and shape consumption choices in prosocial directions (Maeng et al., 2013).

The theoretical tension regarding whether social crowding functions as a catalyst for avoidance or a facilitator of affiliation remains unresolved. We argue that this inconsistency persists because prior studies have largely treated crowding as a monolithic stressor, failing to account for the roles of the sharing-target (close friends vs. strangers) and the underlying psychological states induced by the degree of environmental crowding. Specifically, social crowding often induces physiological and psychological strain, prompting individuals to seek relational security by ‘sharing-in’ with close friends to mitigate perceived threats. Conversely, non-social crowding, characterized by a lack of social presence, can induce feelings of isolation or boredom, which may trigger exploratory interactions, specifically ‘sharing-out’ with strangers, to alleviate the resulting psychological vacuum. Thus, the present research proposes that social crowding is not a determinant of uniform negative or positive outcomes; rather, it acts as an antecedent that directs consumer choices toward either relational consolidation (‘sharing-in’) or exploratory social engagement (‘sharing-out’), depending on the crowding-induced psychological needs and the availability of suitable relational targets.

### 2.2. Shared Consumption

Shared consumption refers to an emerging consumption mode mediated by sharing platforms, wherein consumers exchange access rights to temporarily acquire goods or services without transferring full ownership (Rudmin, 2016). This form of consumption is both common and socially embedded, as illustrated by everyday behaviors such as borrowing a painkiller from a coworker or accepting a mint from a fellow passenger (Lteif et al., 2024). Conceptually, shared consumption can be differentiated into sharing-in, which occurs within close interpersonal relationships, and sharing-out, which involves interactions with socially distant others, such as strangers (Boothby et al., 2016). Sharing-in is grounded in intimacy maintenance, reciprocal trust, emotional security, and relationship reinforcement. Sharing-out is grounded in weak-tie engagement, exploratory social contact, novelty seeking, and openness to socially distant others. Increasingly, public infrastructure facilitates shared consumption by enabling access to communal resources, such as free sunscreen dispensers, thereby addressing issues of resource scarcity. Beyond its functional benefits, shared consumption carries important social implications, fostering interpersonal connection and reinforcing social norms (Taylor & Noseworthy, 2021). Building on this perspective, the present research examines how social crowding shapes shared consumption behaviors, with particular attention to how it influences individuals’ relational orientation toward sharing with close friends versus strangers.

Prior research distinguishes sharing-in from sharing-out by emphasizing differences in interpersonal dynamics and individual predispositions (see Table A2 in Appendix B). Sharing-in is typically embedded within close relationships, such as friendships, familial ties, or romantic partnerships, where individuals exhibit stronger motivations to share and higher levels of relational intimacy (Enestrom et al., 2023; Snyder et al., 2024). Such behaviors reinforce social closeness, trust, and mutual responsiveness (Garcia-Rada & Kim, 2021; E. R. Sun & Jakubiak, 2024). In contrast, sharing-out often involves lower levels of psychological involvement and empathy, as individuals tend to exhibit weaker evaluative and emotional responses when sharing experiences with strangers (Boothby et al., 2017). Nevertheless, emerging evidence suggests that sharing with strangers can, under certain conditions, intensify emotional experiences and facilitate social connection (Li et al., 2021), and repeated shared interactions may foster cooperation and prosocial behavior even in the absence of prior relational ties (Wolf & Tomasello, 2020). Individual differences, including traits such as altruism and social capital, further shape individuals’ willingness to share with socially distant others (Obrenovic et al., 2020). Despite these valuable insights, empirical evidence regarding the specific antecedents that shape individuals’ preferences for these distinct shared consumption modes remains scant. Crucially, the vast majority of prior research has adopted the perspective of non-psychosocial environmental factors, focusing strictly on variables such as time scarcity, physical proximity, sibling relationship, or attachment anxiety. The potential impact of psychosocial environmental factors remains heavily under-explored. Addressing this gap, the present research investigates how social crowding differentially influences sharing-in and sharing-out, thereby advancing understanding of shared consumption as a fundamentally relational process.

## 3. Theoretical Framework and Hypotheses Development

### 3.1. Social Crowding and Shared Consumption

Social crowding refers to a situation in which a large density of individuals occupies a constrained physical space, such as a subway car during peak hours or a bustling retail store (Maeng et al., 2013). Beyond its physical properties, social crowding serves as a prominent social cue that profoundly influences individuals’ cognition, behavioral motivations, and interpersonal dynamics. Prior research indicates that in consumption settings, social crowding heightens human-to-human connectivity, thereby evoking a fundamental need for safety and social connectedness (Huang et al., 2018; Z. Sun et al., 2026). Furthermore, empirical studies confirm that social crowding reinforces emotional bonds with close friends, romantic partners, brands, and established social networks, thereby driving interpersonal communication and word-of-mouth, an effect that is particularly pronounced within stable, existing relationships (Gomillion et al., 2017; Consiglio et al., 2018). Scholars have also demonstrated that social crowding enhances perceived interpersonal intimacy (Kennedy et al., 2009), suggesting that crowded environments amplify individuals’ desire for intimacy and psychological security. Given that sharing-in provides individuals with deeper relational closeness and emotional support, whereas sharing-out offers a greater sense of novelty and fresh experiences (Garcia-Rada & Kim, 2021), it stands to reason that social crowding encourages consumers to prioritize sharing with close friends.

Conversely, non-social crowding refers to scenarios where a sparse number of individuals occupy a physical space, such as an uncrowded beach or a sparsely populated supermarket (Huang et al., 2018). Studies have found that non-social crowding alleviates anxiety and social tension, fostering an atmosphere conducive to initiating conversations with strangers (J. Zhu et al., 2023). Simultaneously, non-social crowding enhances individuals’ motivation to explore their external environment and elevates their need for novelty and fresh experiences (Kennedy et al., 2009), thereby increasing their willingness to engage in novel social activities (Hou et al., 2021). Scholars have also confirmed that non-social crowding, such as retail stores with low foot traffic, similarly heightens individuals’ desire for novelty, rendering them more inclined to engage in sharing behaviors (Du & Liu, 2025; Zhong & Wang, 2025). Furthermore, sharing-out caters to individuals’ higher curiosity and need for novelty, whereas inner-sharing serves the opposite psychological function (Boothby et al., 2016). Synthesizing these insights, non-social crowding encourages consumers to prioritize sharing with strangers.

**H1.**  *Social crowding increases individuals’ preference for sharing with close friends (i.e., sharing-in), whereas non-social crowding enhances their preference for sharing with strangers (i.e., sharing-out).*

### 3.2. The Mediating Role of Psychological Distance

Psychological distance is defined as an egocentric subjective perception reflecting how close or far an object or event is relative to the self (Trope & Liberman, 2010). Existing literature reveals that higher levels of social crowding lead to a significantly shorter psychological distance between individuals (Kirk & Rifkin, 2022). According to Construal Level Theory (CLT), a reduction in psychological distance facilitates interpersonal connection (Trope et al., 2007). Particularly when processing interpersonal information, shortened psychological distance intensifies the need for intimate relationships (Xia et al., 2025), which subsequently elevates individuals’ desire for belongingness and relational attachment (Liu et al., 2020; Williams et al., 2024). Because sharing-in inherently satisfies these heightened needs for intimacy and attachment (Enestrom et al., 2023; Yang et al., 2024), psychological distance serves as a critical underlying mechanism. Synthesizing these insights, social crowding reduces psychological distance between individuals, which in turn drives a stronger preference for the consumption of sharing-in. In contrast, prior literature suggests that non-social crowding (i.e., higher non-social crowding) increases the psychological distance between individuals (Kirk & Rifkin, 2022). According to Construal Level Theory (CLT), an expanded psychological distance leads to more abstract perceptions of others, thereby encouraging more exploratory and novel social tendencies (Trope et al., 2007; Barkan, 2024). When psychological distance is enlarged, individuals’ pursuit of novelty and fresh experiences expands, prompting them to try new modes of social interaction and proactively reach out to socially distant individuals (Sprecher et al., 2023; Jiang et al., 2024). Crucially, sharing-out (e.g., sharing with strangers) effectively satisfies individuals’ desire for novelty and exploratory motives (So et al., 2019; Hall et al., 2023). Synthesizing these insights, non-social crowding enlarges psychological distance, which in turn drives a stronger preference for the consumption of sharing-out.

**H2.**  *Psychological distance mediates the impact of social crowding on shared consumption.*

### 3.3. The Moderating Role of Resource Mindset

Resource mindset refers to individuals’ subjective construal of resource availability, encompassing both scarcity and abundance orientations (Mehta & Zhu, 2016). Scarcity heightens security and relational efficiency concerns, making close-tie sharing more attractive, whereas abundance supports exploratory motives and relative openness to strangers (Liang et al., 2023; M. Zhu & Ratner, 2015). According to scarcity theory, when individuals perceive resources as limited, they develop a scarcity mindset; this mindset heightens their sensitivity to resource utilization and prompts them to adopt more prudent strategies in resource allocation (Shah et al., 2012, 2015; Hamilton et al., 2019). Within this context, sharing with close others becomes a relationally efficient means of maximizing resource value. Scarcity also amplifies the perceived importance of close relationships, fostering a heightened need for security, affiliation, and mutual support (Mehta & Zhu, 2016; Luo et al., 2025). In socially crowded environments, these affiliative motivations are further reinforced, as proximity to others enhances feelings of belonging and interpersonal closeness (Maeng et al., 2013), thereby promoting sharing behaviors within established relationships (Garcia-Rada & Kim, 2021; Liang et al., 2025). In contrast, in non-socially crowded contexts, reduced interpersonal salience may weaken feelings of belonging and diminish the motivation to share with close others (Wicklund, 1974; Kennedy et al., 2009). Accordingly, under a scarcity mindset, social crowding is expected to strengthen individuals’ preference for sharing-in.

Conversely, according to scarcity theory, an abundance mindset reflects the cognitive perception that resources are plentiful and unlimited (Mehta & Zhu, 2016; Liang et al., 2023); this perception mitigates concerns regarding resource depletion and enhances individuals’ willingness to explore (Shah et al., 2012, 2015). Under such conditions, individuals are more likely to engage in outward-oriented behaviors, including interactions with socially distant others (Gao et al., 2024). Resource abundance has been linked to a greater desire for novelty, autonomy, and diverse social experiences, which can facilitate sharing with strangers (Hebrok & Boks, 2017). In socially crowded environments, increased opportunities for interaction may further enhance individuals’ sense of social connectedness and community engagement, thereby encouraging sharing-out behaviors, such as providing information or resources to unfamiliar others (Li et al., 2021; Neville et al., 2022). In contrast, in non-socially crowded contexts, the combination of low social stimulation and resource sufficiency may reduce motivation for social engagement, leading to lower levels of sharing with strangers (O’Guinn et al., 2015; Chen & Ryu, 2024). Therefore, under an abundance mindset, social crowding is expected to promote a preference for sharing-out by encouraging exploratory and outward-oriented social engagement.

**H3.**  *Resource mindset moderates the impact of social crowding on shared consumption.*

**H3a.**  *Under a scarcity mindset, social crowding strengthens individuals’ preferences for sharing-in.*

**H3b.**  *Under an abundance mindset, social crowding strengthens individuals’ preferences for sharing-out.*

**Figure 1:**
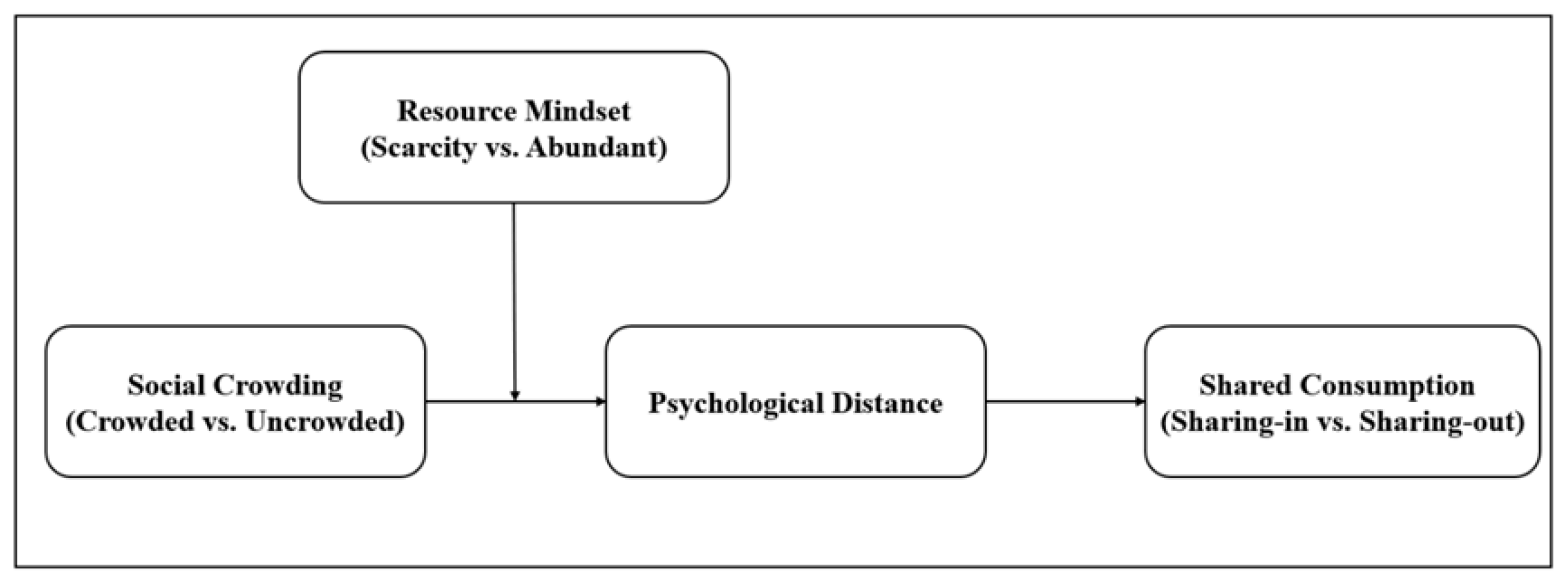
Theoretical Framework.

To empirically test this theoretical framework, we propose four formal experimental studies (see Figure 1).

## 4. Study Overview

To test our research hypotheses, we conducted a package of four studies comprising one field experiment and three scenario-based experiments. Study 1 was conducted in a real-world university cafeteria, adopting a field-based design that relied on participants’ self-reported preferences for sharing with close friends versus sharing with strangers. This field setup established high ecological validity, confirming the main effect of social crowding on sharing consumption in a natural setting and establishing its real-world relevance. Addressing the limitations of potential confounding variables and restricted internal validity in Study 1, Study 2 enhanced internal validity by utilizing dynamic video stimuli of streets with varying crowding levels within a simulated retail scenario (tent-sharing decisions), while rigorously testing the mediating role of psychological distance. To rule out potential visual confounds associated with video content, Study 3 employed a static avatar paradigm with strictly matched background population densities and introduced resource mindset as a moderating variable. Through a refined two-factor design, Study 3 further strengthened internal validity while delineating the boundary conditions of the effect. Finally, to evaluate the cross-context and cross-product generalizability, Additional Study 1 (see Appendix I) manipulated crowding via beach photographs and examined sunscreen-sharing behaviors. Complementing the first three studies, this supplementary experiment extended our findings from dining and outdoor gear to personal care consumables, thereby enhancing the overall external validity of our research. A comprehensive overview of all studies is summarized in Table 1 and Table A3 (see Appendix C).

We have reported all manipulations, sample sizes, measures, and data exclusions for our studies in the manuscript and Appendix A, Appendix B, Appendix C, Appendix D, Appendix E, Appendix F, Appendix G, Appendix H, Appendix I and Appendix J. Study materials, demographic data, additional analyses, and supplemental studies are available in Appendix D, Appendix E, Appendix I and Appendix J. All data are publicly available on the Open Science Framework (OSF): https://osf.io/mkr7v/?view_only=16f03ef5a2c14382b03a2e1d39d9e633 (accessed on 4 April 2026).

## 5. Study 1

Study 1 aimed to examine Hypothesis H1, which predicts that social crowding increases individuals’ propensity for close friends, whereas non-social crowding enhances the propensity for strangers. The study was conducted in a real-world setting, a university cafeteria, using public hand sanitizer as the behavioral measure of shared consumption under varying crowding conditions. Study 1 provides naturalistic evidence rather than isolated causal evidence. Conducted in a real-world setting, it measured participants’ preferences for sharing with close friends versus sharing with strangers based on participants’ self-reports.

### 5.1. Design and Participants

Study 1 lasted 21 days and was conducted between 22 September and 13 October 2025. A total of 130 Chinese participants (M*_Age_* = 26.43; SD = 7.54; 58.2% female; Table 2) from Eastern China were recruited. G*Power 3.1 calculations indicated a minimum sample size of 128 for a medium effect size (*d* = 0.5) at 80% power, *α* = 0.05. Data were collected in the university cafeteria of a teacher-training institution in eastern China. We recruited participants via a random intercept method at the entrances and dining areas of restaurants. We targeted a minimum sample of 128 and slightly oversampled to account for potential exclusions. The study was approved by the university’s ethics committee.

### 5.2. Procedure

**Figure A1:**
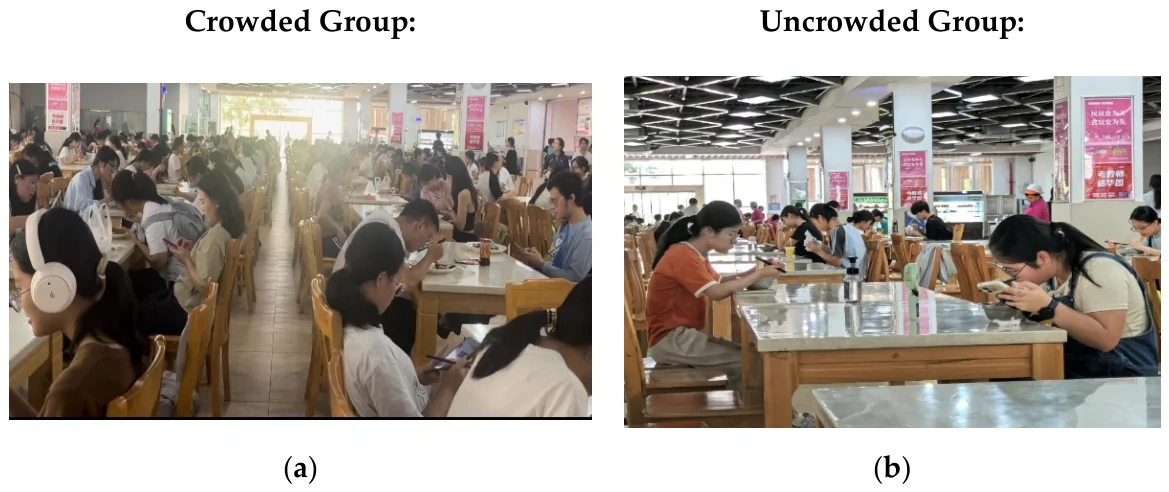
Experimental images in Study 1. (**a**) Photograph of the crowded condition used in Study 1; (**b**) Photograph of the uncrowded condition used in Study 1.

**Figure A2:**
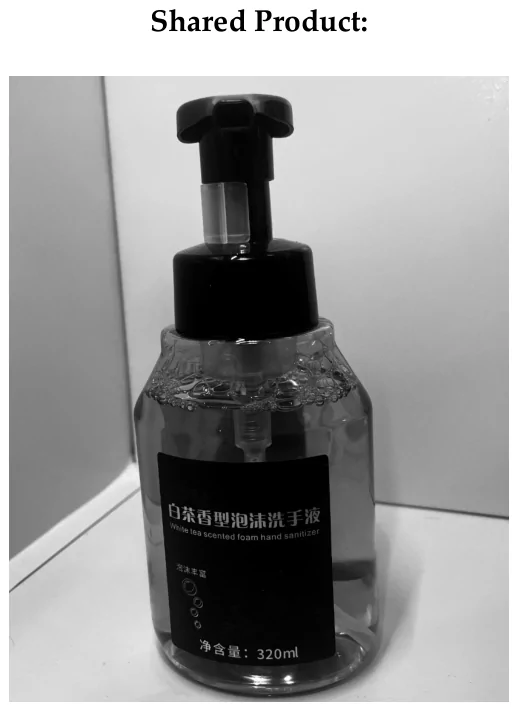
Public hand sanitizer used as the shared product in Study 1.

Study 1 used a two-condition between-subjects design (crowded vs. uncrowded) and was implemented entirely offline in this naturalistic setting. Crowd levels were determined by observation during distinct time periods, and participants were randomly selected accordingly: the crowded condition during peak lunch hours (11:00–12:30; see Figure A1a in Appendix F) and the uncrowded condition during off-peak hours (9:00–10:30; see Figure A1b in Appendix F). A bottle of public hand sanitizer was placed on each dining table (see Figure A2 in Appendix F). To prevent participants from guessing the true purpose of the experiment, all study participants were informed that the research focused on food waste.

Participants rated perceived crowding using two items: “To what extent is the dining environment crowded at this moment?” and “How do you feel in such an environment?” (1 = not crowded at all; 7 = very crowded; α = 0.978, *CR* = 0.978, *AVE* = 0.958; Maeng et al., 2013). Subsequently, participants were instructed to use the hand sanitizer and then respond to a survey item measuring their preference for sharing with close friends versus sharing with strangers: “If you were to use this product after your meal, with whom would you be more willing to share it?” (1 = completely with strangers; 7 = completely with close friends; Boothby et al., 2017). Control variables included product familiarity and potential confounds such as emotional state (irritated–calm; α = 0.847, *CR* = 0.840, *AVE* = 0.736; Watson et al., 1998), arousal (sluggish–tense; α = 0.757, *CR* = 0.759, *AVE* = 0.611; Bradley & Lang, 1994), adaptation to crowding, and need to belong (α = 0.806, *CR* = 0.805, *AVE* = 0.675; Moreland & Levine, 1988). Finally, demographic information was collected.

### 5.3. Results

#### 5.3.1. Manipulation Checks

An independent samples *t*-test showed that participants in the crowded condition (M*_Crowded_* = 6.22, SD = 0.51) reported significantly higher perceptions of crowding than those in the uncrowded condition (M*_Uncrowded_* = 1.98, SD = 0.56), t (128) = 45.25, *p* < 0.001, 95% CI [4.05, 4.42], *d* = 7.94. This confirms the successful manipulation of social crowding.

#### 5.3.2. Correlation Analysis

Table A7 (see Appendix F) reported the bivariate correlations among social crowding, shared consumption, and the remaining study variables. Results indicate a strong and statistically significant positive association between perceived social crowding and shared consumption (*r* = 0.778, *p* < 0.01). In contrast, shared consumption does not display significant correlations with any of the other variables included in the analysis.

#### 5.3.3. Willingness to Share

**Figure 2:**
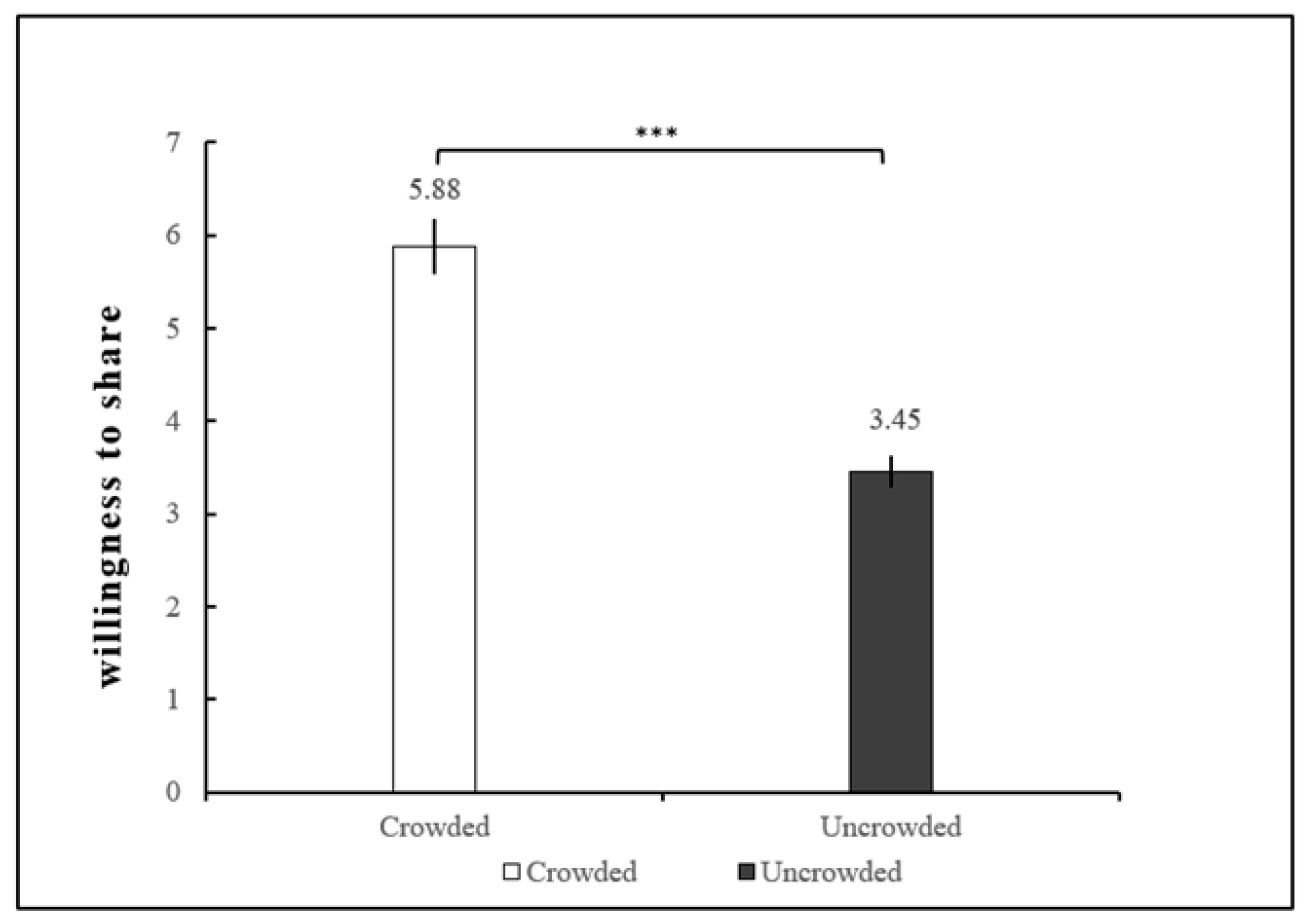
Willingness to share (Study 1). Note: *** *p* < 0.001.

An independent samples *t*-test indicated that participants in the crowded condition (M*_Crowded_* = 5.88, SD = 0.60) were significantly more willing to share with close friends (sharing-in) than those in the uncrowded condition (M*_Uncrowded_* = 3.45, SD = 1.10), t (128) = 15.59, *p* < 0.001, 95% CI [2.12, 2.74], *d* = 2.74. Conversely, participants in the uncrowded condition showed greater willingness to share with strangers (sharing-out). These findings support Hypothesis H1 (see Figure 2).

#### 5.3.4. Analysis of Control Variables

An independent samples *t*-test revealed no significant differences across the social crowding and non-social crowding groups in emotional state (irritated–calm; t (128) = 1.26, *p* = 0.21, 95% CI [−0.14, 0.61]), arousal (sluggish–tense; t (128) = 0.05, *p* = 0.96, 95% CI [−0.31, 0.32]), need to belong (t (128) = 0.89, *p* = 0.37, 95% CI [−0.20, 0.52]), and adaptation to crowding (t (128) = 0.33, *p* = 0.74, 95% CI [−0.31, 0.52]).

## 6. Study 2

Study 2 tested Hypothesis H2, which posits that psychological distance mediates the relationship between social crowding and shared consumption. By employing dynamic video stimuli and a shopping mall consumption context, this study enhanced ecological validity and provided a more immersive test of whether social crowding shapes sharing behavior through shifts in perceived interpersonal proximity and social connectedness.

### 6.1. Design and Participants

Study 2 lasted 30 days and took place between 1 and 30 October 2025. To expand the scope of the experiment, 200 participants were recruited via Credamo (credamo.com) from Eastern China, each compensated 1 RMB. Then, 52 participants who did not meet the experimental requirements were excluded from the analyses. The exclusion criteria included random careless responses and elimination by attention check items. Finally, a total of 148 Chinese participants (M*_Age_* = 31.97; SD = 8.17; 47.3% female; see Table 3) were recruited. G*Power 3.1 indicated a minimum of 128 participants was required for medium effect detection (*d* = 0.5) at 80% power, *α* = 0.05. A between-subjects design (Social Crowding: crowded vs. uncrowded) was used online. The study was approved by the university’s ethics committee.

### 6.2. Procedure

**Figure A3:**
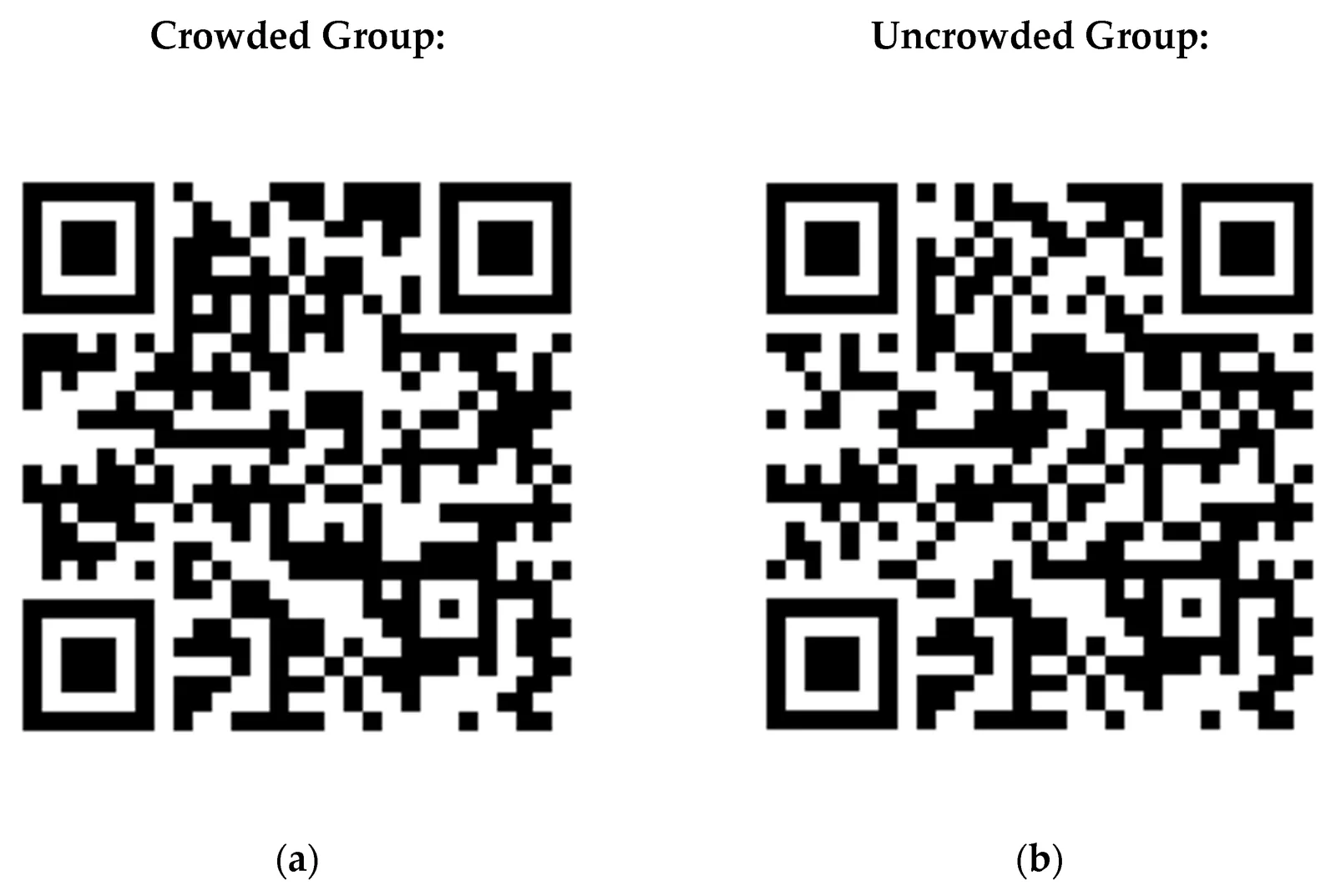
Experimental stimuli videos in Study 2. (**a**) The video content is a crowded commercial street; (**b**) The video content is an uncrowded commercial street. Note: Please scan the QR code to watch the video. (**a**) Video URL: https://youtu.be/zkXvhzqtm9U (accessed on 4 April 2026); (**b**) Video URL: https://youtu.be/JaiBu9mefZc (accessed on 4 April 2026).

Social crowding was manipulated using video stimuli. Participants watched a video and imagined themselves in the depicted setting, then described it in terms of crowd size, interpersonal distance, and personal space. Study 2 explains why video-based stimuli are appropriate for social crowding research: videos capture motion, density, interpersonal spacing, and the experiential flow of crowded environments more realistically than static verbal descriptions. The crowded condition featured a densely populated scene (see Figure A3a in Appendix G); the uncrowded condition showed a nearly empty street (see Figure A3b in Appendix G). Perceived crowding was measured using two items adapted from Huang et al. (2018): “How do you feel about the environment shown in the video?” and “To what extent is the environment in the video crowded?” (1 = Not at all crowded; 7 = Very crowded; α = 0.971, *CR* = 0.971, *AVE* = 0.944).

**Figure A4:**
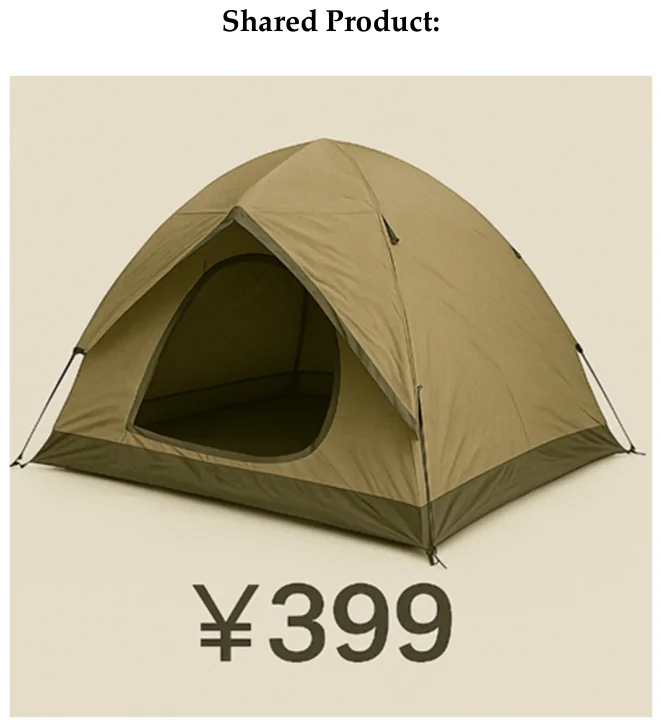
Image of the shared product used in Study 2 (tent rental price: RMB 399).

Next, participants were presented with the following scenario: “Imagine you are going camping and need to rent a tent (e.g., Figure A4 in Appendix G). The tent can accommodate several people, but due to high demand, only one tent is left, and you must share it with others.” They then responded to three items assessing preference for sharing with close friends versus strangers: “With whom will you share the camping equipment rented from this store?” “With whom would you prefer to share your tent?” and “Who are you interested in sharing your tent with?” (1 = Only with strangers; 7 = Only with close friends; α = 0.932, *CR* = 0.936, *AVE* = 0.830), adapted from Nie et al. (2022).

Psychological distance was measured with three items adapted from Bar-Anan et al. (2006): “I feel very close to this person,” “I often communicate with this person,” and “I am willing to share personal matters with this person” (1 = Strongly disagree; 7 = Strongly agree; α = 0.954, *CR* = 0.956, *AVE* = 0.880). This scale was selected because it provides a precise and quantifiable operationalization of psychological distance within social contexts; it effectively captures the nuanced shifts in the perceived bond between the self and others, thereby reflecting the relational changes prompted by the environment. Consistent with the framework of psychological distance (Bar-Anan et al., 2006), we operationalize reduced psychological distance through measures of interpersonal closeness, as a higher degree of closeness reflects a shorter perceived psychological distance between the self and others. Following Pieters et al. (1999), we controlled for video visual quality, color, and appeal (α = 0.516, *CR* = 0.562, *AVE* = 0.300). While the focal constructs in our study demonstrated acceptable levels of reliability and validity, the video-quality control variable exhibited lower psychometric properties. Consequently, this variable is treated strictly as a statistical covariate rather than a core theoretical construct. We report its descriptive statistics to acknowledge its presence in the model, but we caution against interpreting its specific effects beyond its role as a control for potential exogenous variance, emotional state (unhappy–happy; α = 0.834, *CR* = 0.830, *AVE* = 0.710), and arousal (drowsy–alert; α = 0.805, *CR* = 0.834, *AVE* = 0.715), and potential alternative mechanisms such as sense of control (Hock & Bagchi, 2018; α = 0.911, *CR* = 0.913, *AVE* = 0.839), and sense of threat (Ma et al., 2024). Demographic information was collected at the end of the study.

### 6.3. Results

#### 6.3.1. Manipulation Checks

An independent samples *t*-test confirmed the effectiveness of the social crowding manipulation. Participants in the crowded condition (M*_Crowded_* = 6.25, SD = 0.65) reported significantly greater perceived crowding than those in the uncrowded condition (M*_Uncrowded_* = 1.97, SD = 0.81), t (146) = 35.24, *p* < 0.001, 95% CI [4.02, 4.50], *d* = 5.79. These findings indicate that the social crowding manipulation was effective.

#### 6.3.2. Correlation Analysis

Table A9 (see Appendix G) reports the bivariate correlations between social crowding, shared consumption, and the remaining study variables. Results reveal that shared consumption is significantly and positively associated with social crowding (*r* = 0.586, *p* < 0.01) and psychological distance (*r* = 0.586, *p* < 0.01).

#### 6.3.3. Willingness to Share

**Figure 3:**
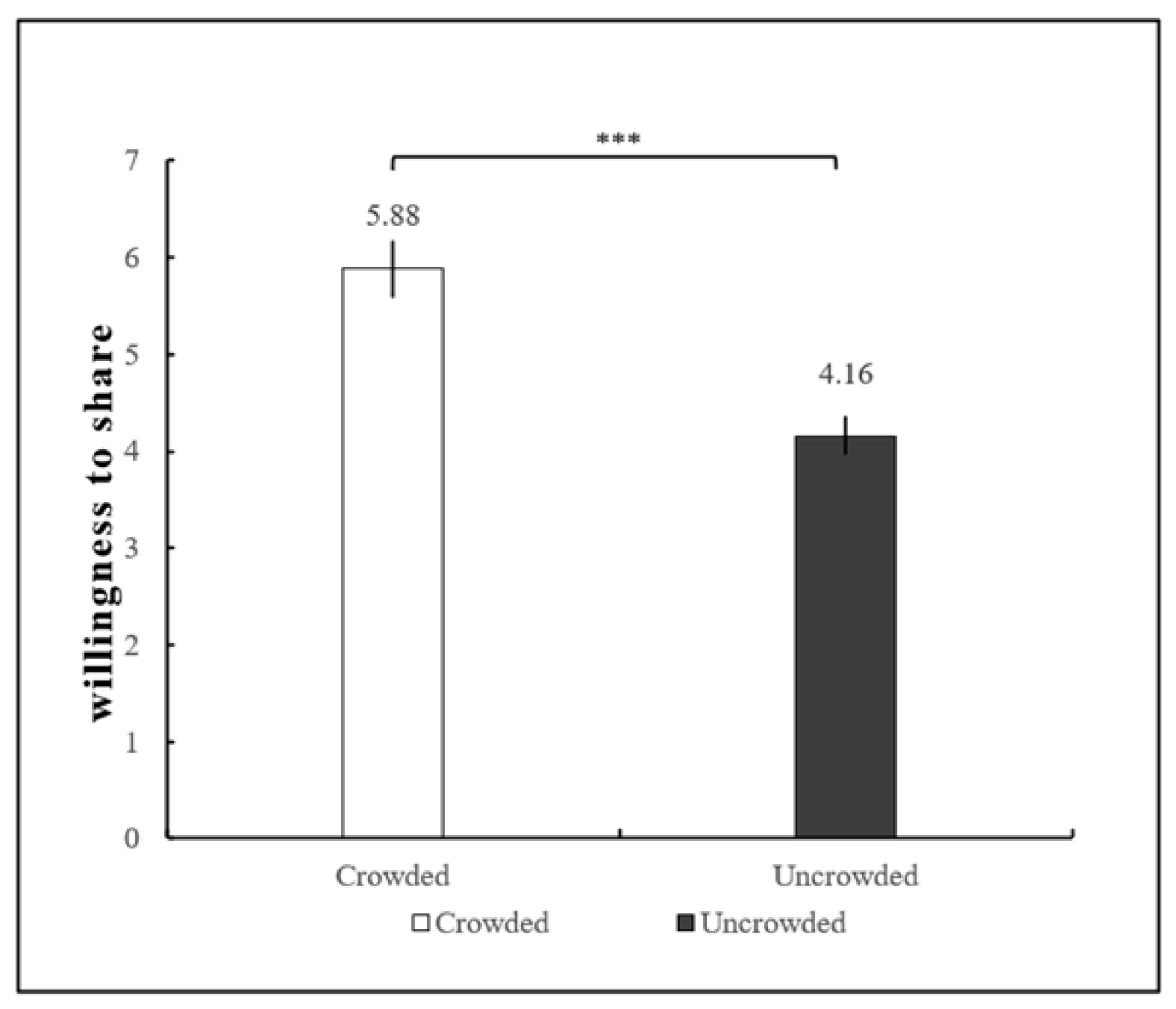
Willingness to Share (Study 2). Note: *** *p* < 0.001.

An independent samples *t*-test revealed that participants in the crowded condition (M*_Crowded_* = 5.88, SD = 0.54) reported significantly greater willingness to share with close friends (i.e., sharing-in) than those in the uncrowded condition (M*_Uncrowded_* = 4.16, SD = 1.56), t (146) = 8.97, *p* < 0.001, 95% CI [1.34, 2.09], *d* = 1.47. Conversely, participants in the uncrowded condition showed higher willingness to share with strangers (i.e., sharing-out). These findings align with Hypothesis H1 (see Figure 3).

#### 6.3.4. Mediation Analysis

A bootstrap mediation analysis (5000 samples) tested whether psychological distance mediated the relationship between social crowding (IV) and willingness to engage in shared consumption (DV). The indirect effect of psychological distance was significant, *β* = 0.29, *SE* = 0.09, 95% CI [0.11, 0.49], as zero was not included in the confidence interval, indicating mediation. Even after accounting for psychological distance, the direct effect of social crowding remained significant, t (145) = 2.60, *p* = 0.01, 95% CI [0.04, 0.31], suggesting partial mediation. These findings support Hypothesis H2.

**Figure 4:**
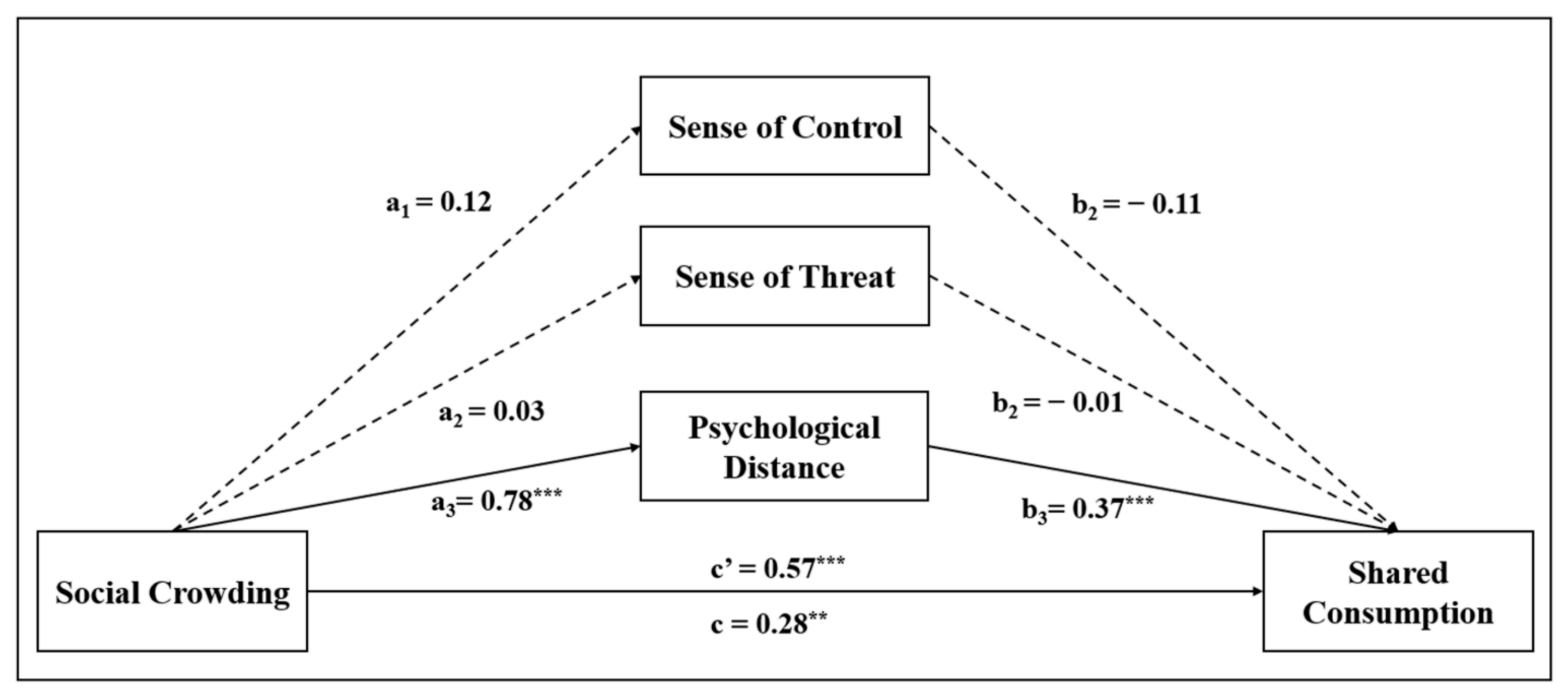
Multiple Mediation Analysis of the Effect of Social Crowding on Shared Consumption (Study 2). Note: ** *p* < 0.01. *** *p* < 0.001.

To further verify that psychological distance was the specific mechanism, a multiple mediation analysis using PROCESS Model 4 (Hayes & Rockwood, 2017) confirmed that only psychological distance mediated the effect of social crowding on shared consumption, while sense of control and sense of threat did not (all 95% CIs included 0; Figure 4).

Hierarchical regression analysis (see Table A10 in Appendix G) further supported the mediating role of psychological distance: social crowding positively predicted shared consumption (*β* = 0.57, *p* < 0.001), positively predicted psychological distance (*β* = 0.78, *p* < 0.001), and maintained a significant effect on shared consumption after including psychological distance (*β* = 0.28, *p* < 0.05).

#### 6.3.5. Alternative Explanation Analysis

To ensure the robustness of our proposed psychological distance mechanism, we included sense of control and perceived threat as competing mediators in Study 2. These constructs were selected because they represent the two most dominant theoretical accounts in prior crowding research. First, social crowding is frequently argued to diminish perceived personal control and environmental autonomy, leading to defensive behavioral adjustments (O’Guinn et al., 2015; Wang & Zhang, 2025). Second, crowding is often conceptualized as a situational stressor that heightens disease transmission concerns and perceived safety threats, thereby inducing social avoidance (Kennedy et al., 2009; Herath et al., 2024). By incorporating these variables, we empirically test whether our observed sharing preferences are a unique function of shifted psychological distance, as predicted by Construal Level Theory (Trope & Liberman, 2010), or if they are merely artifacts of a broader loss of agency or heightened anxiety. Our results demonstrate that, even when accounting for these established pathways, psychological distance remains a significant mediator, suggesting that the relational-sharing framework offers a distinct and parsimonious explanation for consumer behavior in crowded environments.

An independent-samples *t*-test was conducted. The results showed that, for psychological distance, participants in the crowded condition (M*_Crowded_* = 5.70, SD = 1.09) perceived significantly greater closeness than those in the uncrowded condition (M*_Uncrowded_* = 2.35, SD = 1.20), t (146) = 17.77, *p* < 0.001, 95% CI [2.98, 3.73], *d* = 2.92. For sense of control, participants did not differ significantly in perceived control, t (146) = 1.107, *p* = 0.06, 95% CI [−0.22, 0.79]. There was no significant difference in perceived threat between conditions, t (146) = 0.44, *p* = 0.66, 95% CI [−0.47, 0.74].

#### 6.3.6. Analysis of Control Variables

An independent samples *t*-test revealed no significant differences across the crowded and uncrowded groups in video visual quality, color, and appeal (t (146) = 0.03, *p* = 0.98, 95% CI [–0.33, 0.34]), emotional state (unhappy–happy; t (146) = 1.58, *p* = 0.12, 95% CI [–0.06, 0.50]), and arousal (drowsy–alert; t (146) = 0.31, *p* = 0.76, 95% CI [–0.18, 0.25]).

## 7. Study 3

Study 3 examined Hypothesis H3, which predicted that under a scarcity mindset, social crowding would heighten preference for sharing-in, whereas under an abundance mindset, social crowding would promote sharing-out. Employing immersive virtual avatar-based scenarios and a realistic tent rental task, this study sought to enhance ecological validity and deliver rigorous empirical validation for H3.

### 7.1. Design and Participants

Study 3 lasted 30 days and was conducted between 6 October and 4 November 2025. To expand the scope of the experiment, 352 participants were recruited via Credamo (credamo.com) from Southeast China and received 2 RMB each. Then, 60 participants who did not meet the experimental requirements were excluded from the analyses. The exclusion criteria included random careless responses and elimination by attention check items. Finally, a total of 292 Chinese participants (M*_Age_* = 32.09; SD = 8.38; 52.4% female; see Table 4) completed the study. Based on G*Power 3.1, 179 participants were needed to detect a medium effect (*f* = 0.25) with 80% power, *α* = 0.05. The study used a 2 (Social Crowding: crowded vs. uncrowded) × 2 (Resource Mindset: scarcity vs. abundance) between-subjects design, conducted online with post-completion compensation. The study was approved by the university’s ethics committee.

### 7.2. Procedure

First, we manipulated resource mindset through autobiographical recall tasks (see Appendix H). We chose this method because resource mindset is fundamentally a subjective construal of resource sufficiency, rather than a purely objective reflection of one’s financial or temporal status. By asking participants to recount personal experiences characterized by scarcity or abundance, we were able to activate personally meaningful resource appraisals. Crucially, this episodic retrieval approach ensures that the manipulated mindset is distinct from the subsequent social crowding scenario, thereby minimizing demand effects and procedural confounding. In the scarcity condition, participants described a personal experience involving resource deprivation, such as lacking money, time, or essential goods, and reflected on the associated emotions. In the abundance condition, participants described an experience of resource surplus using the same procedure. Manipulation effectiveness was assessed with two items adapted from Roux et al. (2015): “I feel that I do not have enough resources” and “I live in a harsh environment” (1 = Strongly disagree, 7 = Strongly agree; α = 0.833, *CR* = 0.847, *AVE* = 0.736).

**Figure A5:**
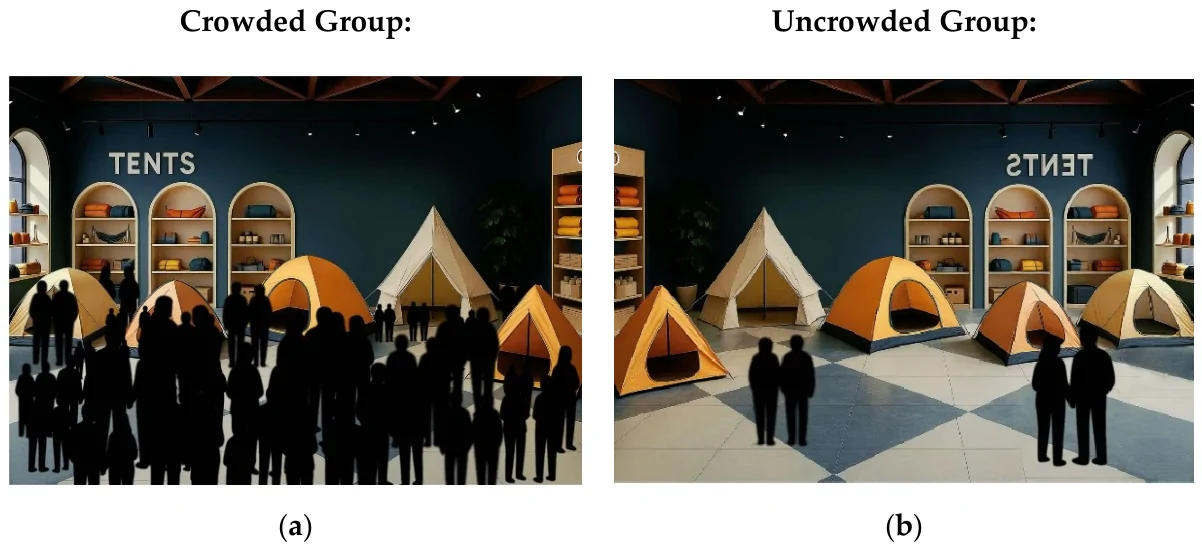
Images of experimental stimuli in Study 3. (**a**) Pictures of the crowded tent store (with virtual characters and backgrounds) were used in Study 3; (**b**) Pictures of the uncrowded tent store (with virtual characters and backgrounds) were used in Study 3.

Second, social crowding was manipulated using images of virtual characters embedded in simulated retail environments. Participants imagined themselves in the depicted scenario: the crowded condition displayed a densely populated tent rental store (see Figure A5a in Appendix H), whereas the uncrowded condition showed a sparsely populated store (see Figure A5b in Appendix H). Effectiveness was assessed with two items: “How do you feel about the environment shown in the image?” and “To what extent is the environment in the image crowded?” (1 = Not at all crowded, 7 = Very crowded; α = 0.969, *CR* = 0.969, *AVE* = 0.939).

**Figure A6:**
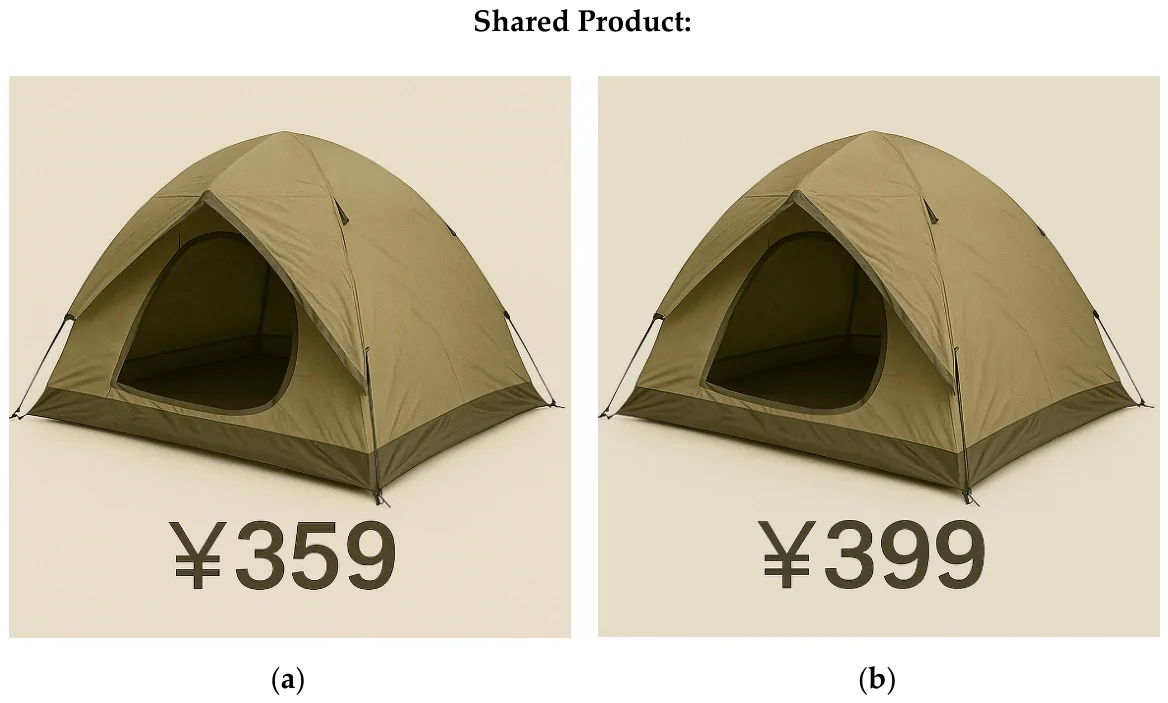
Images of experimental stimuli in Study 3. (**a**) Images of the shared products used in Study 3 (tent prices: RMB 359); (**b**) Images of the shared products used in Study 3 (tent prices: RMB 399).

Next, shared consumption was measured via the scenario: “Imagine you are camping and need to rent a tent (see Figure A6a,b in Appendix H). The tent accommodates multiple people, but due to high demand, only one remains. You must share it with others.” Participants then indicated their preference for sharing with close friends versus strangers: “When renting the product, whom would you prefer to share it with?” (1 = completely with strangers; 7 = completely with close friends). This measure captures participants’ relative preference for the target of sharing (close friends vs. strangers) along a continuum, rather than measuring the absolute willingness to share with a specific individual.

To control for the influence of price difference, participants answered: “When choosing between two tents priced at 399 yuan and 359 yuan, and assuming that you have 400 yuan (or 1000 yuan) available, which tent would you choose? (1 = 359 yuan, 7 = 399 yuan)”, adapted from Janiszewski and Lichtenstein (1999). Participants also rated image quality, clarity, and visual appeal (α = 0.662, *CR* = 0.809, *AVE* = 0.585), as well as emotional state (calm–anxious; α = 0.721, *CR* = 0.723, *AVE* = 0.566) to account for potential confounds. Finally, demographic information was collected.

### 7.3. Results

#### 7.3.1. Manipulation Checks

Social crowding: A 2 (Social Crowding: crowded vs. uncrowded) × 2 (Resource Mindset: scarcity vs. abundance) ANOVA revealed a significant main effect of social crowding on perceived crowding, F (1, 288) = 73.30, *p* < 0.001, *η_p_*^2^ = 0.20. Neither the main effect of resource mindset, F (1, 288) = 0.24, *p* = 0.63, nor the interaction, F (1, 288) = 0.001, *p* = 0.98, was significant. The manipulation was thus deemed effective.

Resource mindset: ANOVA on perceived scarcity showed a significant main effect of resource mindset, F (1, 288) = 38.70, *p* < 0.001, *η_p_*^2^ = 0.12, but a nonsignificant main effect of social crowding, F (1, 288) = 0.96, *p* = 0.33. The interaction was significant, F (1, 288) = 5.82, *p* < 0.05, *η_p_*^2^ = 0.02. Simple effects analyses confirmed that the resource-mindset manipulation remained highly effective across both crowded and uncrowded conditions. While this interaction suggests that the perceived strength of the resource manipulation was slightly qualified by the social density context, the primary directional effect of the mindset induction remained robust and intact within each condition. Thus, we accept the manipulation check as successful while appropriately noting this partial contextual dependency.

#### 7.3.2. Correlation Analysis

Table A12 (see Appendix H) reported the bivariate correlations between social crowding, shared consumption, and the remaining study variables. The results indicate that shared consumption is positively associated with price difference (r = 0.423, *p* < 0.01) and emotional state (calm–anxious) (*r* = 0.157, *p* < 0.01). Nevertheless, shared consumption is negatively associated with Gender (*r* = −0.130, *p* < 0.05).

#### 7.3.3. Moderation Analysis

A 2 (Social Crowding: crowded vs. uncrowded) × 2 (Resource Mindset: scarcity vs. abundance) ANOVA examining the interaction between social crowding and resource mindset on shared consumption revealed a significant main effect of social crowding, F (1, 288) = 6.54, *p* = 0.01, *η_p_*^2^ = 0.02. Participants in the crowded condition (M*_Crowded_* = 6.49, SD = 0.84) showed greater willingness to share with close friends (sharing-in) than those in the uncrowded condition (M*_Uncrowded_* = 6.28, SD = 0.92). The main effect of resource mindset was not significant, F (1, 288) = 0.80, *p* = 0.37. Crucially, the interaction between social crowding and resource mindset was significant, F (1, 288) = 32.48, *p* < 0.001, *η_p_*^2^ = 0.10.

**Figure 5:**
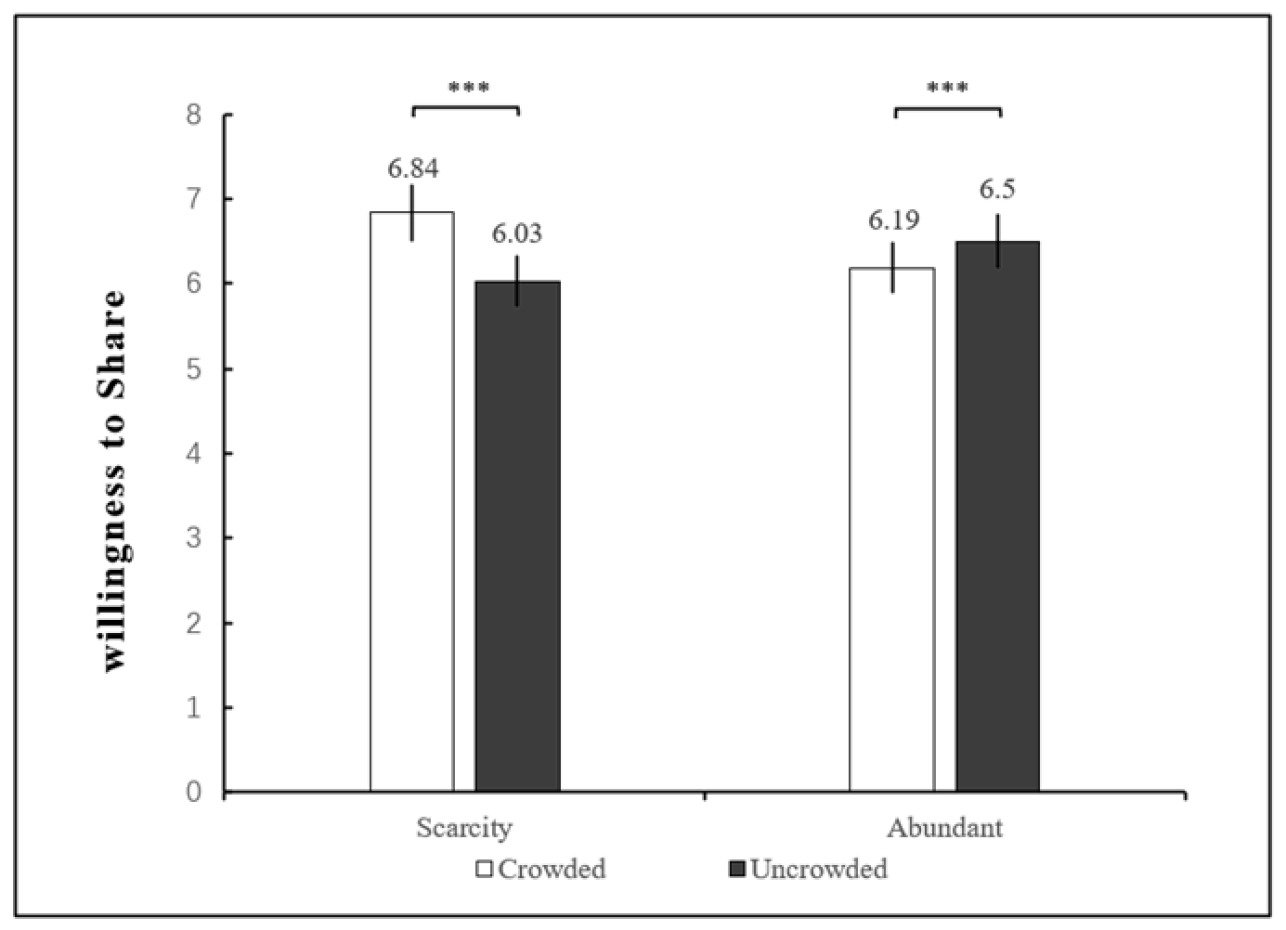
Interaction between Social Crowding and Resource Mindset (Study 3). Note: *** *p* < 0.001.

Under the scarcity mindset, participants in the crowded condition (M*_Crowded_* = 6.84, SD = 0.37) exhibited greater willingness to share with close friends than those in the uncrowded condition (M*_Uncrowded_* = 6.03, SD = 1.11), t (288) = 5.72, *p* < 0.001, 95% CI [0.53, 1.09], *d* = 0.98. Conversely, under the abundance mindset, the crowded condition (M*_Crowded_* = 6.19, SD = 1.01) produced a relative shift away from close-friend-oriented sharing and toward greater openness to stranger-oriented sharing compared with the uncrowded condition (M*_Uncrowded_* = 6.50, SD = 0.64), t (288) = −2.28, *p* = 0.02, 95% CI [−0.57, −0.04], d = 0.37. These findings align with H3 (see Figure 5).

#### 7.3.4. Analysis of Control Variables

Two-factor analysis of variance revealed no significant differences in image quality, clarity, and visual appeal (F (1, 290) = 2.79, *p* = 0.10), emotional state (calm–anxious; F (1, 290) = 0.008, *p* = 0.93), and price difference (F (1, 290) = 0.098, *p* = 0.76).

## 8. General Discussion

While prior research has produced seemingly contradictory evidence regarding the impact of social crowding, often reporting increased avoidance, stress (O’Guinn et al., 2015; Kennedy et al., 2009), and territoriality alongside findings of heightened affiliation and social warmth (Maeng et al., 2013; Consiglio et al., 2018), our findings reconcile these discrepancies by demonstrating that crowding effects are contingent upon the relational target and the underlying resource mindset. Rather than denying the existence of avoidance behaviors, we propose that such negative responses are likely triggered when strangers, perceived threats, or involuntary density are salient. In contrast, our framework highlights that under conditions of relational security, social crowding paradoxically fosters close-tie ‘sharing-in.’ Furthermore, we refine the understanding of social facilitation by showing that while affiliation is a core outcome of crowding, its target is selective, favoring close friends over strangers. Conversely, we argue that the reduced tension associated with non-social crowding (i.e., low-density environments) supports a different psychological state, one that facilitates exploratory interactions with unfamiliar others and produces a relative shift toward ‘sharing-out’ behaviors. By integrating these relational and contextual boundary conditions, our research provides a more nuanced resolution to the traditional ‘stress-versus-affiliation’ debate in crowding literature.

This study provides empirical evidence supporting how social crowding influences individuals’ preferences for different modes of shared consumption (sharing-in vs. sharing-out), indicating that social crowding promotes sharing with close friends, while non-social crowding facilitates sharing with strangers. This effect is mediated by psychological distance. Resource mindset moderates this effect. Specifically, individuals under social crowding are more likely to prefer sharing with close friends, whereas participants in non-social crowding are relatively more open to sharing with strangers (Study 1 and Additional Study 1). This effect is mediated by psychological distance (Study 2). We further elucidated that resource mindset serves as a moderating factor: in conditions of scarcity, social crowding amplifies sharing-in, whereas in conditions of abundance, it facilitates sharing-out (Study 3). Together, these findings extend current understanding of how crowding shapes relationally situated sharing decisions.

### 8.1. Theoretical Implications

First, this study deepens the social crowding literature while extending collaborative consumption theory. While prior research on social crowding has predominantly focused on its negative outcomes, such as heightened time scarcity (Garcia-Rada & Kim, 2021), adverse physiological stress (Z. Zhang et al., 2023), or altered human-chatbot interactions (J. Zhu et al., 2023), our work demonstrates that social crowding can actively foster positive relational engagement. Specifically, we analyze two distinct modes within collaborative consumption theory (sharing-in with close friends vs. sharing-out with strangers) under varying density settings. By utilizing social crowding (vs. non-social crowding) as an innovative entry point to explore preferences for these sharing modes, this study broadens the applicability of collaborative consumption theory across distinct spatial and human density contexts, complementing existing research on relational sharing.

Second, this study advances Construal Level Theory (CLT) and psychological distance theory by elucidating cognitive distance as a pivotal underlying mechanism. Psychological distance and its associated construal levels have been shown to influence perceptions of relational boundaries (Argo et al., 2005) and consumer choices across diverse domains, including shared accommodation (S. Zhang et al., 2023), identity-relevant consumption (Zheng et al., 2023), and e-commerce (Liu et al., 2020). Building on this foundation, our research demonstrates that social crowding reduces perceived psychological distance, activating a lower, concrete construal level focused on close interpersonal bonding, thereby enhancing feelings of belonging and promoting sharing-in. Conversely, non-social crowding increases perceived distance, shifting consumers to a higher, abstract construal level centered on personal autonomy, which encourages sharing-out. Integrating CLT into collaborative consumption extends the theory’s applicability to interpersonal relationship dynamics and highlights its explanatory power in relational choices. Finally, this research enriches Scarcity Theory by identifying resource mindset as a key boundary condition. Prior research suggests that interpersonal proximity and perceived resource availability jointly dictate responses to crowded environments (L. Cheng et al., 2023). Individuals with a scarcity mindset tend to prioritize safety, security, and social affiliation (Neville et al., 2022), whereas those with an abundance mindset emphasize autonomy and exploration. Consistent with this perspective, our findings reveal that under a scarcity mindset, social crowding strengthens preferences for sharing-in, whereas under an abundance mindset, social crowding increases tendencies toward sharing-out. Incorporating resource mindset enriches Scarcity Theory within consumer behavior, demonstrating how situational density and cognitive resource mindsets interactively govern collaborative consumption.

### 8.2. Practical Implications

First, our findings demonstrate that social crowding systematically shapes consumers’ preferences for different forms of shared consumption (i.e., sharing-in versus sharing-out), providing actionable guidance for businesses that rely on collaborative consumption models. Specifically, service providers can tailor sharing formats according to customer density. For example, in shared restaurants (Study 1), managers may emphasize close-circle sharing options, family-style meal bundles, and private-table hygiene kits during peak hours to accommodate consumers’ stronger preference for sharing with familiar others while simultaneously improving service efficiency and alleviating discomfort associated with crowded environments. During off-peak periods, restaurants may instead introduce optional communal tables or shared appetizer pairings for solo diners to encourage interactions among unfamiliar customers and reduce the psychological emptiness often associated with sparsely populated environments. Likewise, retailers (Studies 2 and 3) may promote group bundles or friend-oriented product trial experiences during busy shopping periods, whereas community-based product demonstrations or stranger-friendly trial events may be more effective when customer density is relatively low.

Second, by identifying psychological distance as the underlying mechanism, our research offers practical implications for tourism operators and sharing platforms seeking to encourage collaborative consumption. Because social crowding reduces perceived psychological distance, tourism destinations may provide private group equipment rentals or shared resource packages (e.g., sunscreen or outdoor equipment) during crowded travel seasons to capitalize on consumers’ greater willingness to share with familiar companions. Conversely, during less crowded periods, operators may facilitate voluntary gear-pooling programs or resource-sharing stations for solo travelers, thereby encouraging interactions among strangers. Similarly, sharing platforms can dynamically recommend private-group bookings in high-density locations while promoting verified stranger-matching services in low-density destinations or co-rental settings, where consumers may be more receptive to establishing new social connections.

Finally, our findings reveal that resource mindset serves as an important boundary condition, suggesting that managers should consider consumers’ perceived resource availability when designing shared consumption strategies. Rather than applying a uniform sharing strategy, businesses should adapt collaborative consumption initiatives to both the social environment and consumers’ resource mindset. For instance, accommodation-sharing platforms and ride-sharing services may place greater emphasis on family- or friend-oriented group discounts during periods when consumers experience greater resource scarcity, as familiar sharing partners may provide stronger feelings of security and resource preservation. By contrast, when consumers perceive resources to be relatively abundant, platforms may encourage stranger-based matching or carpooling services by highlighting opportunities for social interaction, convenience, and efficient resource utilization. Such adaptive strategies may enhance consumer acceptance while maximizing the effectiveness of shared consumption programs across different market conditions.

## 9. Limitations and Future Research

This study investigates how social crowding (e.g., overcrowded environments) influences individuals’ preferences for different modes of shared consumption (sharing-in vs. sharing-out). However, several limitations of the present research remain to be acknowledged.

First, relying exclusively on Chinese samples warrants caution regarding cross-cultural generalizability; future studies should test whether these findings hold in individualistic cultures. Second, from a methodological standpoint, our empirical evidence relies primarily on cross-sectional experimental designs, capturing short-term snapshot responses. Future research should employ longitudinal designs or field tracking to examine how social crowding dynamically shapes shared consumption over time.

Second, regarding the underlying mechanism, this study explicitly examined only one mediator—psychological distance. While psychological distance offers a powerful explanatory framework for mapping interpersonal boundaries under varying density, other competing or parallel mechanisms may also account for these effects. Future research should explore potential alternative pathways, such as interpersonal trust, perceived contamination, social identity, territoriality, or perceived ownership, to provide a more comprehensive picture of how crowding environments shape relational consumption decisions.

Third, from a measurement perspective, our empirical studies relied primarily on self-reported preferences and hypothetical sharing intentions within controlled scenario experiments. Although these methods effectively isolate causal mechanisms, they may not fully reflect real-world decision-making. Future scholars are strongly encouraged to deploy field experiments and unobtrusive observational studies to capture consumers’ actual, consequential sharing behaviors in naturalistic environments.

Fourth, future research can extend our framework by explicitly distinguishing between human and spatial density, incorporating nuanced relationship types (e.g., coworkers or acquaintances), and exploring consumption dynamics in digital sharing environments. To enhance experimental precision, we recommend that subsequent studies systematically control for contextual factors such as consumption purpose, time pressure, hunger levels, group size, and initial social status (e.g., solo vs. accompanied). Finally, we advocate for replicating our findings across diverse, low-risk shared consumption contexts—such as portable chargers, umbrellas, picnic mats, or public hygiene supplies—to bolster ecological validity and test the generalizability of our model in mundane, real-world interactions.

## Figures and Tables

**Table 1:** Overview of Studies.

Study	Objective	Hypotheses Tested	Manipulation	Dependent Variable	Incremental Contribution
Study 1	Initial field evidence	H1	Naturally crowded vs. uncrowded cafeteria	Shared consumption preference for public sanitizer	Ecological validity in a real service setting
Study 2	Mechanism test	H1, H2	Crowded vs. uncrowded street video	Shared consumption preference for tent	Tests mediation and alternative mechanisms
Study 3	Boundary-condition test	H3, H3a, H3b	Virtual crowding x scarcity/abundance recall	Shared consumption preference for tent	Tests resource mindset as moderator
Additional Study 1	Conceptual replication	H1	Crowded vs. uncrowded beach setting	Shared consumption preference for sunscreen	Extends context to tourism/public shared resource

**Table 2:** Descriptive statistics for Study 1.

Study 1						
N = 130, 58.2% Females, M*_Age_* = 26.43 Years, SD = 7.54						
	M (SD)		*df*	*p*	95% CI	*d*
Variables	Crowded	Uncrowded				
Social Crowding	6.22(0.51)	1.98(0.56)	t (128) = 45.25	<0.001	[4.05, 4.42]	7.94
Shared Consumption	5.88(0.60)	3.45(1.10)	t (128) = 15.59	<0.001	[2.12, 2.74]	2.74
Emotional state (irritated–calm)	2.95 (1.19)	2.72 (0.95)	t (128) = 1.26	=0.21	[−0.14, 0.61]	-
Arousal (sluggish–tense)	2.85 (0.98)	2.85 (0.82)	t (128) = 0.05	=0.96	[−0.31, 0.32]	-
Need to Belong	2.92 (1.24)	2.76 (0.78)	t (128) = 0.89	=0.37	[−0.20, 0.52]	-
Adaptation to Crowding	2.80 (1.13)	2.74 (1.00)	t (128) = 0.33	=0.74	[−0.31, 0.43]	-
Gender	3.09 (2.01)	3.40 (1.96)	t (128) = −0.88	=0.38	[−1.00, 0.38]	-
Age	27.48 (8.19)	25.38 (6.74)	t (128) = 1.59	=0.11	[−0.51, 4.69]	-
Education	3.46 (0.87)	3.23 (0.63)	t (128) = 1.73	=0.09	[−0.03, 0.49]	-
Personal Income	2.43 (1.29)	2.05 (1.17)	t (128) = 1.79	=0.08	[−0.04, 0.81]	-
Occupation	2.95 (0.96)	2.85 (0.91)	t (128) = 0.66	=0.51	[−0.21, 0.43]	-

Notes: Statistics in parentheses are standard deviations.

**Table 3:** Descriptive statistics for Study 2.

Study 2						
N = 148, 47.3% Females, M*_Age_* = 31.97 Years, SD = 8.17						
	M (SD)		*df*	*p*	95% CI	*d*
Variables	Crowded	Uncrowded				
Social Crowding	6.24(0.65)	1.97(0.81)	t (146) = 35.24	<0.001	[4.02, 4.50]	5.79
Shared Consumption	5.88(0.54)	4.16(1.56)	t (146) = 8.97	<0.001	[1.34, 2.09]	1.47
Psychological Distance	5.70 (1.09)	2.35 (1.20)	t (146) = 17.77	<0.001	[2.98, 3.73]	2.92
Emotional state (unhappy–happy)	3.73 (0.88)	3.51 (0.84)	t (146) = 1.58	=0.12	[–0.06, 0.50]	-
Arousal (drowsy–alert)	3.85 (0.67)	3.82 (0.66)	t (146) = 0.31	=0.76	[–0.18, 0.25]	-
Sense of Control	3.32 (1.65)	3.03 (1.46)	t (146) = 1.107	=0.06	[−0.22, 0.79]	-
Sense of Threat	4.50 (1.85)	4.36 (1.88)	t (146) = 0.44	=0.66	[–0.47, 0.74]	-
Video visual quality, color, and appeal	2.90 (1.05)	2.89 (1.01)	t (146) = 0.03	=0.98	[−0.33, 0.34]	-
Gender	2.84 (2.01)	2.95 (2.01)	t (146) = −0.33	=0.74	[−0.76, 0.55]	-
Age	33.23 (8.89)	30.70 (7.22)	t (146) = 1.90	=0.06	[−0.10, 5.16]	-
Education	2.88 (0.66)	3.00 (0.50)	t (146) = −1.27	=0.21	[−0.31, 0.07]	-
Occupation	1.61 (1.20)	1.66 (1.19)	t (146) = −0.28	=0.78	[−0.44, 0.33]	-
Personal Income	3.70 (1.40)	3.47 (1.40)	t (146) = 1.00	=0.32	[−0.22, 0.68]	-

Notes: Statistics in parentheses are standard deviations.

**Table 4:** Descriptive statistics for Study 3.

Study 3					
N = 292, 52.4% Females, M*_Age_* = 32.09 Years, SD = 8.38					
		M (SD)		*df*	*p*
Variables		Crowded	Uncrowded		
Social Crowding		5.39 (1.06)	4.10 (1.47)	F (1, 288) = 73.30	<0.001
Resource Mindset	Scarcity Mindset	5.03 (1.14)		F (1, 288) = 38.70	<0.001
	Abundance Mindset	4.11 (1.37)			
Shared Consumption	Scarcity Mindset	6.84 (0.37)	6.03 (1.11)	t (134) = 5.72	<0.001
	Abundance Mindset	6.19 (1.01)	6.50 (0.64)	t (154) = −2.28	=0.02
Price difference	Scarcity Mindset	4.18 (2.63)	3.48 (2.54)	t (134) = 1.56	=0.12
	Abundance Mindset	4.21 (2.49)	4.86 (2.58)	t (154) = −1.61	=0.11
Emotional state (calm–anxious)	Scarcity Mindset	3.95 (0.78)	3.87 (0.76)	t (134) = 0.61	=0.54
	Abundance Mindset	3.83 (0.68)	3.85 (0.78)	t (154) = 0.16	=0.87
Image quality, clarity, and visual appeal	Scarcity Mindset	3.85 (0.92)	3.67 (0.92)	t (134) = 1.15	=0.25
	Abundance Mindset	3.76 (0.98)	3.57 (0.97)	t (154) = 1.21	=0.23
Gender	Scarcity Mindset	3.24 (2.00)	3.00 (2.02)	t (134) = 0.68	=0.50
	Abundance Mindset	3.05 (2.01)	3.10 (2.01)	t (154) = 0.16	=0.87
Age	Scarcity Mindset	33.53 (8.85)	31.53 (9.56)	t (134) = 1.08	=0.28
	Abundance Mindset	32.69 (8.16)	30.96 (6.94)	t (154) = 1.43	=0.16
Education	Scarcity Mindset	2.99 (0.61)	2.93 (0.61)	t (134) = 0.56	=0.57
	Abundance Mindset	2.95 (0.62)	2.96 (0.59)	t (154) = −0.13	=0.90
Occupation	Scarcity Mindset	1.74 (1.35)	1.87 (1.22)	t (134) = 0.60	=0.55
	Abundance Mindset	1.56 (1.17)	1.76 (1.27)	t (154) = 0.98	=0.33
Personal Income	Scarcity Mindset	3.65 (1.46)	3.16 (1.58)	t (134) = 1.86	=0.07
	Abundance Mindset	3.54 (1.42)	3.49 (1.49)	t (154) = 0.22	=0.83

Notes: Statistics in parentheses are standard deviations.

**Figure A7:**
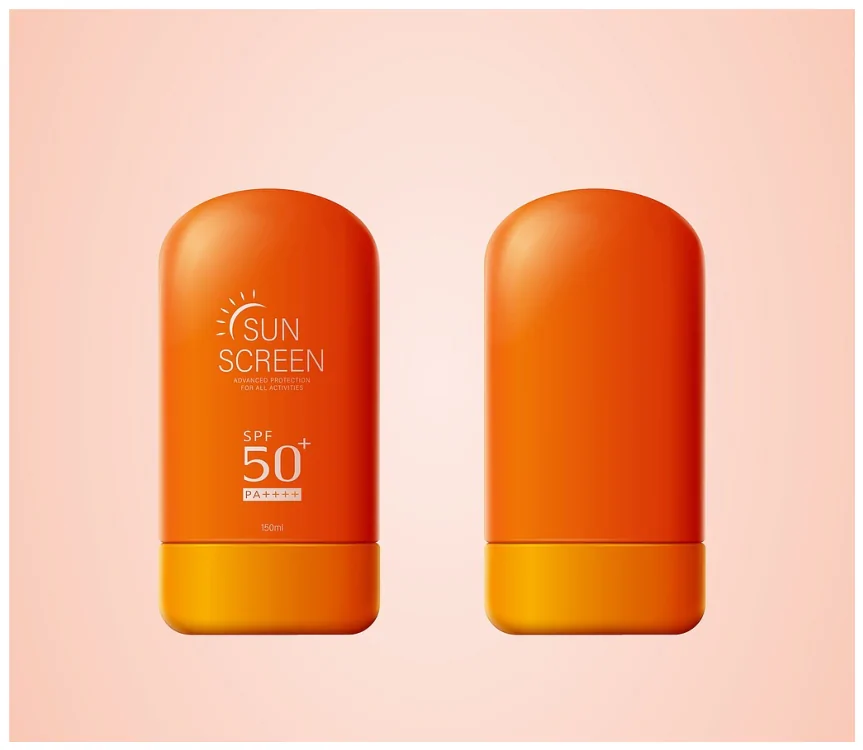
Pictures of the shared product (private sunscreen) were used in Additional Study 1.

**Figure A8:**
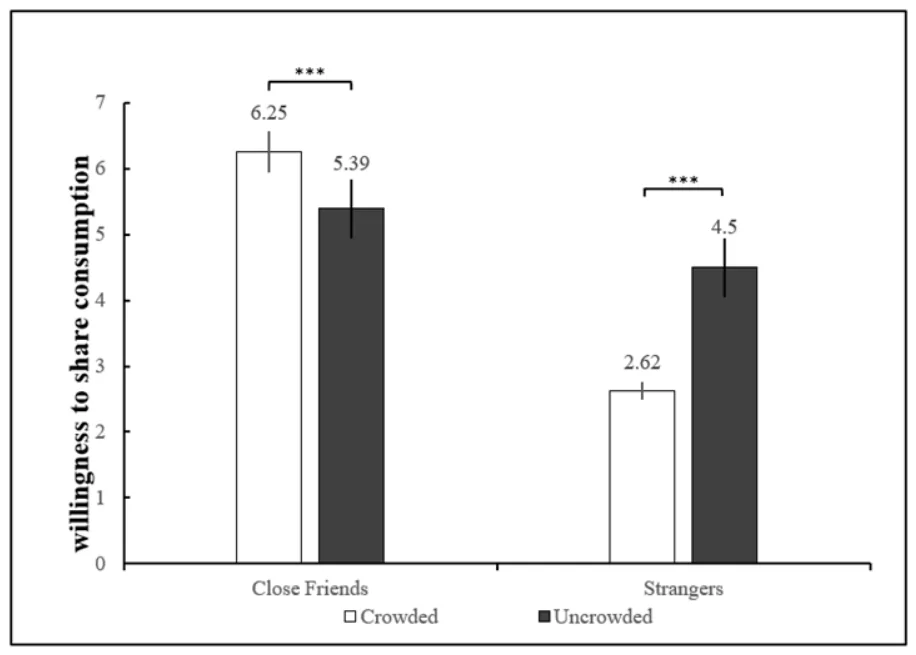
Willingness to Share (Additional Study 1). Note: *** *p* < 0.001.

**Figure A9:**
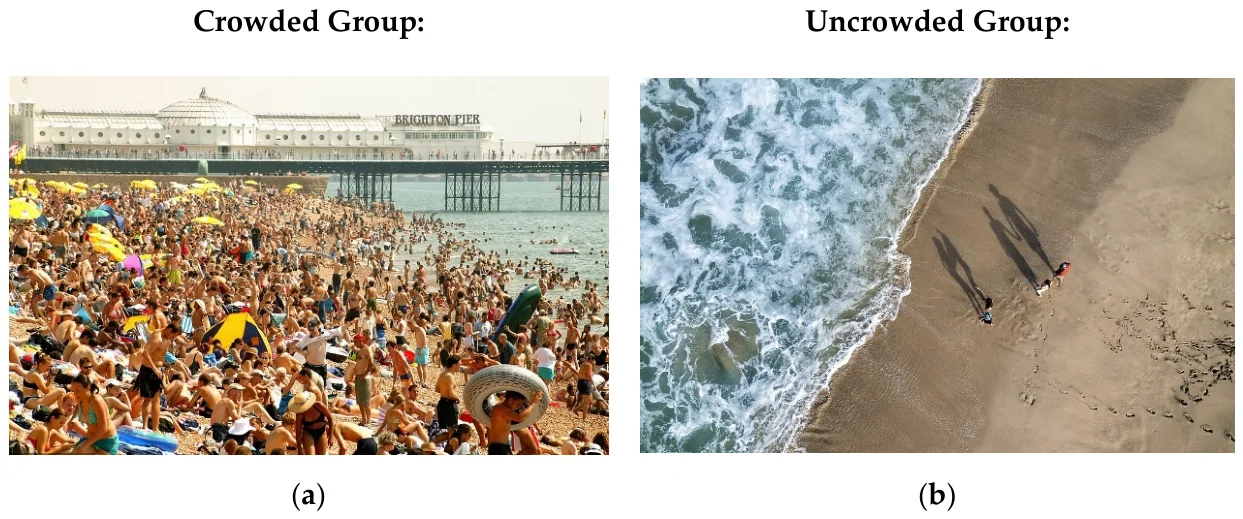
Images of experimental stimuli in Additional Study 1. (**a**) Pictures of crowded beaches were used in Additional Study 1; (**b**) Pictures of uncrowded beaches were used in Additional Study 1.

## Data Availability

The data that support the findings of this study are available online at https://osf.io/mkr7v/overview?view_only=9578954579e04c03b9ee6cd2ec887881 (accessed on 4 April 2026).

## Appendix A

**Table A1:** Research on Social Crowding.

Research Area	Research Perspective	Source	Dependent Variable(s)	Relevant Findings
Physiological Health	Non-shared perspective	Herath et al. (2024)	COVID-19 incidence rate	The effects of urban density and household overcrowding on COVID-19 infections vary with the local context.
		Kennedy et al. (2009)	Amygdala activation in humans	Individuals with complete amygdala damage exhibit a diminished sense of personal space, which can significantly impact their emotional state.
		Z. Zhang et al. (2023)	Human physiological stress response	Overcrowding can strongly elicit human physiological stress responses. Additional contributing factors include the presence of large gatherings, recreational facilities, vehicles, and bicycles.
Psychological Level		O’Guinn et al. (2015)	Product valuation	As the social density of a given space increases, individuals’ ability to accurately infer the subjective social class and income of others within that space diminishes.
		Huang et al. (2018)	Brand attachment	A crowded environment can enhance individuals’ attachment to a brand.
		Wang and Zhang (2025)	Knowledge withholding	Social crowding increases individuals’ perceived loss of knowledge power, reduced knowledge-sharing efficacy, and heightened concern about information control while reducing task visibility, and these factors indirectly contribute to increased knowledge withholding.
		Wicklund (1974)	Psychological reactance	When individuals perceive a loss of personal freedom and spatial autonomy, they experience heightened discomfort and anxiety.
Behavioral Preferences		Maeng et al. (2013)	Consumer choice	Social crowding not only leads to greater accessibility of safety-related constructs but also results in greater preference for safety-oriented options, being more receptive to prevention-framed messages, and being more risk-averse with real money gambles.
		Consiglio et al. (2018)	Word-of-mouth, information sharing	Increased social density can enhance the likelihood of information sharing, although individuals’ long-term need for control may attenuate this effect.
		Peng et al. (2024)	Preference for natural products	Social crowding stimulated tourists to exhibit a stronger preference for natural products as a means of coping with ontological security threats.
Behavioral Preferences	Non-shared perspective	J. Zhu et al. (2023)	Preference for chatbots	In a low social crowding environment, consumers prefer chatbots with a social-oriented (vs. task-oriented) conversational style, while in a high social crowding environment, consumers prefer a task-oriented (vs. social-oriented) conversational style, and warmth and competence mediate these effects.
	**Shared perspective**	**This Research**	**Shared consumption**	**Individuals in social crowding are more likely to share-in, while those in non-social crowding are more likely to share-out.**

## Appendix B

**Table A2:** Research on Shared Consumption.

Domain	Research Perspective	Source	Independent Variable(s)	Relevant Findings
Sharing-in	Non-structural interpersonal factors	Snyder et al. (2024)	Attachment anxiety	Individuals characterized by anxiety and dependency are more likely to seek excessive attention, comfort, assistance, and emotional support from close others, particularly when disclosing negative personal experiences.
		Bedrov and Gable (2024)	Sharing and receiving secrets	Close relationships foster greater disclosure of personal secrets, with sharing and receiving intimate information strongly linked to heightened relational intimacy and increased social utility.
		Younkin et al. (2021)	Sibling relationship	Attending college with siblings strengthens relational bonds, enhancing willingness to share daily experiences and deepening connectedness within sibling relationships.
		Enestrom et al. (2023)	Physical proximity	The physical proximity between partners is positively associated with greater intimacy and higher levels of mutual relationship satisfaction.
		Garcia-Rada and Kim (2021)	Time scarcity	When time resources are limited, individuals are more likely to share extraordinary experiences with close others as a means of maintaining and strengthening their relationships.
		E. R. Sun and Jakubiak (2024)	Avoidant attachment	Individuals high in attachment avoidance are more inclined to share positive experiences with close others as a way to showcase personal competence while minimizing emotional vulnerability.
Sharing-out		Li et al. (2021)	Mode of sharing travel experiences	When individuals share positive travel experiences with strangers in a private setting and receive favorable evaluations, they are likely to experience enhanced positive emotions.
		Wolf and Tomasello (2020)	Co-viewing videos	After engaging in activities such as watching movies or reading books with an unfamiliar adult, individuals become more willing to share with them.
		Lteif et al. (2024)	Type of sharing	Sharing products with strangers reduces perceived product efficacy, particularly among individuals with strong self–brand associations, highlighting how interpersonal sharing contexts shape consumer–brand relationships and product evaluations.
		Obrenovic et al. (2020)	Altruism	Individuals who identify with an altruistic group orientation are more inclined to share knowledge—particularly tacit knowledge—with strangers.
		Boothby et al. (2017)	Type of co-experiencer	Sharing visual experiences with close others enhances emotional responses, including liking and perceived realism, whereas sharing with strangers attenuates these effects, underscoring relational context in shaping experiential evaluations.
Sharing-out	Non-structural interpersonal factors	Boothby et al. (2016)	Shared experience/psychological distance	When individuals perceive a high degree of psychological closeness, their experiences tend to be amplified, whereas psychological distance attenuates this effect.
**Sharing-in/Sharing-out**	**Structural social factors**	**This Research**	**Social crowding**	**Individuals in social crowding are more likely to share-in, while those in non-social crowding are more likely to share-out.**

## Appendix C

**Table A3:** Overviews of Studies.

Studies	Design	Social Crowding (IV)	Shared Consumption (DV)	Research Hypothesis
Study 1 (N = 130)	Social Crowding: (Crowded vs. Uncrowded)	Peak lunch hours (11:00–12:30) versus off-peak hours (9:00–10:30)	Use of public hand sanitizer	H1: Social crowding increases sharing-in, whereas non-social crowding increases sharing-out.
Study 2 (N = 148)	Social Crowding: (Crowded vs. Uncrowded)	Crowded pedestrian video vs. video of a nearly empty street	Tent rental	H2: Psychological distance mediates the effect of social crowding on shared consumption.
Study 3 (N = 292)	2 (Social Crowding: Crowded vs. Uncrowded) × 2 (Resource Mindset: Scarcity vs. Abundance)	Simulated crowding via virtual scenes and characters	Tent rental	H3: Resource mindset moderates the relationship between social crowding and shared consumption.
Additional Study 1 (N = 272; see Appendix I)	2 (Social Crowding: Crowded vs. Uncrowded) × 2 (Shared Community: Close Other vs. Stranger)	Crowded beach vs. Uncrowded beach	Sunscreen sharing	H1: Social crowding increases sharing-in, while non-social crowding increases sharing-out.

## Appendix D

**Table A4:** Summary of Studies.

Studies	Main Purposes	Context	Findings	Sample Size and Main Results
Study 1 (Field study at a normal university canteen in Eastern China, from 22 September to 13 October 2025)	Designed to test Hypothesis 1 (H1): Social crowding promotes inward sharing-in, while non-social crowding facilitates outward sharing-out.	Field study	The results demonstrate that social crowding increases sharing-in, whereas non-social crowding shifts preference relatively toward sharing with strangers in shared-consumption contexts.	N = 130 Crowded: M*_Crowded_* = 5.88, SD = 0.60 Uncrowded: M*_Uncrowded_* = 3.45, SD = 1.10 t (128) = 15.59, *p* < 0.001, 95% CI [2.12, 2.74], *d* = 2.74.
Study 2 (Credamo, Eastern China, conducted between 1 and 30 October 2025)	This study tests Hypothesis 2 (H2): Psychological distance mediates the effect of social crowding on shared consumption.	Online	The results not only reaffirm the causal effect of social crowding on shared consumption, but also identify psychological distance as a key mediator in this relationship.	N = 148 Crowded: M*_Crowded_* = 5.88, SD = 0.54 Uncrowded: M*_Uncrowded_* = 4.16, SD = 1.56 t (146) = 8.97, *p* < 0.001, 95% CI [1.34, 2.09], *d* = 1.47. Indirect effect = 0.29, SE = 0.09, 95% CI [0.11, 0.49]
Study 3 (Credamo, Southeast China, conducted between 6 October and 4 November)	This study examines the moderating role of resource mindset (abundance vs. scarcity) on collaborative consumption (H3).	Online	Social crowding polarizes shared consumption by resource mindset: it promotes sharing-in during scarcity but facilitates sharing-out under abundance.	N = 292 Scarcity mindset: M*_Crowded_* = 6.84 vs. M*_Uncrowded_* = 6.03 t (288) = 5.72, *p* < 0.001, 95% CI [0.53, 1.09], *d* = 0.98. Abundance mindset: M*_Crowded_* = 6.19 vs. M*_Uncrowded_* = 6.50 t (288) = −2.28, *p* = 0.02, 95% CI [−0.57, −0.04], *d* = 0.37.
Additional Study 1 (Credamo, Southern China, conducted between 6 October and 4 November 2025)	This study examines the nuanced effects of social crowding on shared-consumption patterns.	Online	The results demonstrate that individuals in social crowding exhibit greater willingness for sharing-in, whereas individuals in non-social crowding show stronger willingness for sharing-out	N = 272 Close friends: M*_Crowded_* = 6.25 vs. M*_Uncrowded_* = 5.39 t (268) = 5.51, *p* < 0.001, 95% CI [0.55, 1.17], *d* = 0.94. Strangers: M*_Crowded_* = 2.62 vs. M*_Uncrowded_* = 4.50 t (268) = −8.64, *p* < 0.001, 95% CI [−2.32, −1.45], *d* = −1.48.

## Appendix E

**Table A5:** Demographic Information of Studies.

Item	Category	Study 1 (N = 130)	Study 2 (N = 148)	Study 3 (N = 292)	Additional Study 1 (N = 272)
Gender	Male	43.8%	52.7%	47.6%	49.6%
	Female	56.2%	47.3%	52.4%	50.4%
Age	≤25	60.0%	21.6%	24.7%	20.5%
	25−35	24.6%	52.0%	46.9%	46.0%
	35−45	4.3%	19.0%	20.9%	27.5%
	45−55	13.9%	6.0%	5.1%	4.9%
	≥55	1.5%	1.4%	2.4%	1.1%
Education	High School or Below	0.8%	3.4%	3.4%	0.4%
	Associate Degree	4.6%	9.5%	10.3%	11.0%
	Bachelor’s Degree	64.6%	77.7%	73.6%	75.4%
	Master’s Degree	19.2%	8.8%	12.7%	12.9%
	Doctoral Degree	10.8%	0.7%	0.0%	0.4%
Occupation	Corporate Employee	14.6%	74.3%	71.2%	71.7%
	Self-employed or Business Owner	3.1%	2.7%	2.7%	2.9%
	Student	61.5%	14.2%	14.4%	14.3%
	Teacher	19.2%	2.7%	5.5%	4.0%
	Other	1.5%	6.1%	6.2%	7.0%
Personal Income	3000 RMB or Below	35.4%	10.1%	14.0%	12.9%
	3001 RMB–5000 RMB	30.0%	16.9%	18.8%	11.4%
	5001 RMB–7000 RMB	16.9%	14.9%	11.3%	18.8%
	7001 RMB–9000 RMB	10.8%	20.3%	18.5%	19.1%
	9001 RMB or Above	6.9%	37.8%	37.3%	37.9%

## Appendix F. Materials Supporting Study 1

### Appendix F.1. Manipulation Check Items for Study 1

**Experimental situations (Group 1: Crowded)** 1. To what extent is the dining environment crowded at this moment? 2. How do you feel in such an environment? (1 = not crowded at all; 7 = very crowded)
**Experimental situations (Group 2: Uncrowded)** 1. To what extent is the dining environment crowded at this moment? 2. How do you feel in such an environment? (1 = not crowded at all; 7 = very crowded)

### Appendix F.3. Item Scale

**Table A6:** Measurement Items for Study 1.

Variables	Measurement Items	Factor Loading	*α*	*CR*	*AVE*	References
Social Crowding (IV)	1. To what extent is the dining environment crowded at this moment? 2. How do you feel in such an environment? (1 = not crowded at all; 7 = very crowded).	0.984/0.973	0.978	0.978	0.958	Maeng et al. (2013)
Willingness to share (DV)	If you were to use this product after your meal, with whom would you be more willing to share it? (1 = completely with strangers; 7 = completely with close friends)	-	-	-	-	Boothby et al. (2017)
Emotional state	Please indicate the extent to which you experienced the following emotions in this environment: 1. I felt irritable. 2. I felt calm. (1 = Very slightly or not at all; 7 = Extremely)	0.844/0.872	0.847	0.840	0.736	Watson et al. (1998)
Arousal	Please indicate the extent to which you experienced the following arousal states in this environment: 1. I felt sluggish. 2. I felt tense. (1 = Very slightly or not at all; 7 = Extremely)	0.755/0.808	0.757	0.759	0.611	Bradley and Lang (1994)
Need to Belong	1. I felt a strong sense of belonging with the environment. 2. I felt that I belonged to the group in this environment. (1 = Strongly disagree; 7 = Strongly agree)	0.815/0.828	0.806	0.805	0.675	Moreland and Levine (1988)
Adaptation to Crowding	I did not feel any discomfort in this environment. (1 = Strongly disagree; 7 = Strongly agree)	-	-	-	-	-
Gender	1. Male 5. Female	-	-	-	-	-
Age	___________years	-	-	-	-	-
Education	1. High school or below 2. Junior college 3. Bachelor’s degree 4. Master’s degree 5. Doctoral degree	-	-	-	-	-
Occupation	1. Employee 2. Self-employed/Business owner 3. Student 4. Teacher 5. Other	-	-	-	-	-
Personal Income	1. Below ¥3000 2. ¥3001–¥5000 3. ¥5001–¥7000 4. ¥7001–¥9000 5. Above ¥9000	-	-	-	-	-

### Appendix F.4. Correlation Analysis

**Table A7:** Correlation Analysis in Study 1.

	V1	V2	V3	V4	V5	V6	V7	V8	V9	V10	V11
V1: Social Crowding	1										
V2: Shared Consumption	0.778 **	1									
V3: Emotional state (irritated–calm)	0.104	0.089	1								
V4: Arousal (sluggish–tense)	−0.026	0.076	0.673 **	1							
V5: Adaptation to Crowding	−0.013	−0.049	0.494 **	0.515 **	1						
V6: Need to Belong	0.060	0.048	0.543 **	0.553 **	0.519 **	1					
V7: Gender	−0.040	−0.096	0.051	−0.096	−0.031	0.045	1				
V8: Age	0.125	0.094	0.123	0.133	0.053	0.118	−0.090	1			
V9: Education	0.140	0.116	0.018	0.020	0.108	0.153	−0.107	0.622 **	1		
V10: Occupation	0.045	0.092	−0.051	−0.018	0.008	0.024	−0.095	0.182 *	0.626 **	1	
V11: Personal Income	0.145	0.110	0.163	0.178 *	0.101	0.200 *	0.007	0.756 **	0.576 **	0.108	1

*. The correlation is significant at the 0.05 level (two-tailed). **. The correlation is significant at the 0.01 level (two-tailed).

## Appendix G

### Appendix G.1. Manipulation Check Items for Study 2

**Experimental situations (Group 1: Crowded)** *1. Please watch the following video, imagine yourself in the scene, and answer the questions below.* 1. How do you feel about the environment shown in the video? 2. To what extent is the environment in the video crowded? (1 = Not at all crowded; 7 = Very crowded) *2. Imagine you are going camping and need to rent a tent. The tent can accommodate several people, but due to high demand, only one tent is left, and you must share it with others.* 1. With whom will you share the camping equipment rented from this store? 2. With whom would you prefer to share your tent? 3. Who are you interested in sharing your tent with? (1 = Only with strangers; 7 = Only with close friends)
**Experimental situations (Group 2: Uncrowded)** *1. Please watch the following video, imagine yourself in the scene, and answer the questions below.* 1. How do you feel about the environment shown in the video? 2. To what extent is the environment in the video crowded? (1 = Not at all crowded; 7 = Very crowded). *2. Imagine you are going camping and need to rent a tent. The tent can accommodate several people, but due to high demand, only one tent is left, and you must share it with others.* 1. With whom will you share the camping equipment rented from this store? 2. With whom would you prefer to share your tent? 3. Who are you interested in sharing your tent with? (1 = Only with strangers; 7 = Only with close friends)

### Appendix G.3. Item Scale

**Table A8:** Measurement Items for Study 2.

Variables	Measurement Items	Factor Loading	*α*	*CR*	*AVE*	References
Social Crowding (IV)	1. How do you feel about the environment shown in the video? 2. To what extent is the environment in the video crowded? (1 = Not at all crowded; 7 = Very crowded).	0.986/0.957	0.971	0.971	0.944	Huang et al. (2018)
Willingness to share (DV)	1. With whom will you share the camping equipment rented from this store? 2. With whom would you prefer to share your tent? 3. Who are you interested in sharing your tent with? (1 = Only with strangers; 7 = Only with close friends)	0.870/0.934/0.927	0.932	0.936	0.830	Nie et al. (2022)
Psychological distance (M)	1. I feel very close to this person. 2. I often communicate with this person. 3. I am willing to share personal matters with this person. (1 = Strongly disagree; 7 = Strongly agree)	0.911/0.941/0.961	0.954	0.956	0.880	Bar-Anan et al. (2006)
Emotional state	Please indicate the extent to which you experienced the following emotions in this environment: 1. I felt unhappy. 2. I felt happy. (1 = Very slightly or not at all; 7 = Extremely).	0.833/0.852	0.834	0.830	0.710	Watson et al. (1998)
Arousal	Please indicate the extent to which you experienced the following arousal states in this environment: 1. I felt drowsy. 2. I felt alert. (1 = Very slightly or not at all; 7 = Extremely)	0.801/0.888	0.805	0.834	0.715	Bradley and Lang (1994)
Sense of Control	Please indicate the extent to which you agree with the following statements: 1. In such an environment, I felt the situation was beyond my control. 2. In the video setting, I found it difficult to feel in control. (1 = Strongly disagree; 7 = Strongly agree)	0.912/0.920	0.911	0.913	0.839	Hock and Bagchi (2018)
Sense of Threat	Sense of threat (reverse-coded): I did not feel threatened in this environment. (1 = Strongly disagree; 7 = Strongly agree)	-	-	-	-	Ma et al. (2024)
Video visual quality, color, and appeal	1. The visual presentation of the video affected my ability to answer the questions accurately. 2. The colors used in the video are overly intense and visually overwhelming, significantly influencing my judgment. 3. The overall attractiveness of the video is distracting and impairs my ability to concentrate on the questions. (1 = Strongly disagree; 7 = Strongly agree)	0.495/0.577/0.568	0.516	0.562	0.300	Pieters et al. (1999)
Gender	1. Male 5. Female	-	-	-	-	-
Age	___________years	-	-	-	-	-
Education	1. High school or below 2. Junior college 3. Bachelor’s degree 4. Master’s degree 5. Doctoral degree	-	-	-	-	-
Occupation	1. Employee 2. Self-employed/Business owner 3. Student 4. Teacher 5. Other	-	-	-	-	-
Personal Income	1. Below ¥3000 2. ¥3001–¥5000 3. ¥5001–¥7000 4. ¥7001–¥9000 5. Above ¥9000	-	-	-	-	-

### Appendix G.4. Correlation Analysis

**Table A9:** Correlation Analysis in Study 2.

	V1	V2	V3	V4	V5	V6	V7	V8	V9	V10	V11	V12	V13
V1: Social Crowding	1												
V2: Psychological Distance	0.783 **	1											
V3: Shared Consumption	0.565 **	0.586 **	1										
V4: Emotional state (unhappy–happy)	0.155	0.196 *	0.158	1									
V5: Arousal (drowsy–alert)	0.004	0.060	−0.015	0.299 **	1								
V6: Sense of Threat	0.026	0.009	0.023	−0.167 *	−0.112	1							
V7: Sense of Control	0.117	0.087	−0.043	0.128	0.133	−0.223 **	1						
V8: Video visual quality, color, and appeal	0.061	−0.020	−0.010	0.087	0.182 *	0.110	0.214 **	1					
V9: Gender	0.031	−0.020	−0.026	−0.004	0.022	0.078	0.058	0.107	1				
V10: Age	0.152	0.125	0.083	0.101	−0.011	0.161	0.056	−0.056	−0.018	1			
V11: Education	−0.044	−0.130	−0.092	0.075	−0.096	−0.194 *	0.038	0.019	−0.064	−0.218 **	1		
V12: Occupation	−0.003	0.024	−0.019	0.032	0.113	−0.131	0.139	−0.008	0.075	−0.126	−0.091	1	
V13: Personal Income	0.076	0.051	−0.012	0.091	−0.052	0.165 *	−0.043	0.017	−0.176 *	0.483 **	0.218 **	−0.458 **	1

*. The correlation is significant at the 0.05 level (two-tailed). **. The correlation is significant at the 0.01 level (two-tailed).

### Appendix G.5. Mediation Analysis Results for Study 2

**Table A10:** Results for the Mediation Analysis in Study 2.

Variable	Model 1		Model 2		Model 3	
	Shared Consumption		Psychological Distance		Shared Consumption	
	β	t	β	t	β	t
Social Crowding	0.57	8.27 ***	0.78	15.20 ***	0.28	2.60 *
Psychological Distance					0.37	3.51 ***
R-squared	0.32		0.61		0.37	
Adjusted R-squared	0.32		0.61		0.36	
F	68.44 ***		231.073 ***		43.03 ***	

**Note:** Standardized regression coefficients are displayed. * *p* < 0.05. *** *p* < 0.001.

## Appendix H

### Appendix H.1. Manipulation Check Items for Study 3

**Experimental situations (Group 1: Crowded × Scarcity)** *1. Please recall a time when you experienced scarcity, that is, a situation where you felt that resources (such as money, time or products) were lacking. Then, take some time to vividly recall what happened at that time and how you felt being in such a scarce situation. Please describe in detail and write a short paragraph in the box below. Please ensure that your memory clearly reflects a situation involving scarce resources.* I feel that I do not have enough resources I live in a harsh environment (1 = strongly disagree, 7 = strongly agree) *2. Next, please look at this picture, imagine yourself in it and answer the following questions.* How do you feel about the environment shown in the image? To what extent is the environment in the image crowded? (1 = not crowded at all, 7 = very crowded) *3. Imagine that you are camping and need to rent a tent. The tent can accommodate many people, but due to high demand, only one tent remains. You must share it with others.*
**Experimental situations (Group 2: Crowded × Abundance)** *1. Please recall a time when you experienced abundance, that is, a situation where you felt that resources (such as money, time or products) were abundant. Then, take some time to vividly recall what happened at that time and how you felt in that resource-scarce situation. Please describe in detail and write a short paragraph in the box below. Please ensure that your memories are in line with the theme of abundant resources.* I feel that I do not have enough resources I live in a harsh environment (1 = strongly disagree, 7 = strongly agree) *2. Next, please look at this picture, imagine yourself in it and answer the following questions.* How do you feel about the environment shown in the image? To what extent is the environment in the image crowded? (1 = not crowded at all, 7 = very crowded) *3. Imagine that you are camping and need to rent a tent. The tent can accommodate many people, but due to high demand, only one tent remains. You must share it with others.*
**Experimental situations (Group 3: Uncrowded × Scarcity)** *1. Please recall a time when you experienced scarcity, that is, a situation where you felt that resources (such as money, time or products) were lacking. Then, take some time to vividly recall what happened at that time and how you felt being in such a scarce situation. Please describe in detail and write a short paragraph in the box below. Please ensure that your memories are in line with the theme of scarce resources.* I feel that I do not have enough resources I live in a harsh environment (1 = strongly disagree, 7 = strongly agree) *2. Next, please look at this picture, imagine yourself in it and answer the following questions.* How do you feel about the environment shown in the image? To what extent is the environment in the image crowded? (1 = not crowded at all, 7 = very crowded) *3. Imagine that you are camping and need to rent a tent. The tent can accommodate many people, but due to high demand, only one tent remains. You must share it with others.*
**Experimental situations (Group 4: Uncrowded × Abundance)** *1. Please recall a time when you experienced abundance, that is, a situation where you felt that resources (such as money, time or products) were abundant. Then, take some time to vividly recall what happened at that time and how you felt in that resource-abundant situation. Please describe in detail and write a short paragraph in the box below. Please ensure that your memories are in line with the theme of abundant resources.* I feel that I do not have enough resources I live in a harsh environment (1 = strongly disagree, 7 = strongly agree) *2. Next, please look at this picture, imagine yourself in it and answer the following questions.* How do you feel about the environment shown in the image? To what extent is the environment in the image crowded? (1 = not crowded at all, 7 = very crowded) *3. Imagine that you are camping and need to rent a tent. The tent can accommodate many people, but due to high demand, only one tent remains. You must share it with others.*

### Appendix H.3. Item Scale

**Table A11:** Measurement Items for Study 3.

Variables	Measurement Items	Factor Loading	*α*	*CR*	*AVE*	References
Social Crowding (IV)	1. How do you feel about the environment shown in the image? 2. To what extent is the environment in the image crowded? (1 = not crowded at all, 7 = very crowded)	0.968/0.970	0.969	0.969	0.939	Huang et al. (2018)
Willingness to share (DV)	When renting the product, who would you prefer to share it with? (1 = completely with strangers; 7 = completely with close friends)	-	-	-	-	Boothby et al. (2017)
Resource mindset (W)	1. I feel that I do not have enough resources 2. I live in a harsh environment (1 = strongly disagree, 7 = strongly agree)	0.774/0.934	0.833	0.847	0.736	Roux et al. (2015)
Price difference	When choosing between two tents priced at 399 yuan and 359 yuan, and assuming that you have 400 yuan (or 1000 yuan) available, which tent would you choose? (1 = 359 yuan, 7 = 399 yuan)	-	-	-	-	Janiszewski and Lichtenstein (1999)
Emotional state	Please indicate the extent to which you experienced the following emotions in this environment: 1. I felt calm. 2. I felt anxious. (1 = Very slightly or not at all; 7 = Extremely).	0.762/0.742	0.721	0.723	0.566	Watson et al. (1998)
Image quality, clarity, and visual appeal	1. The image colors fail to accurately convey topic-relevant information and lack visual appeal. 2. The image is unclear, lacks sufficient resolution, and contains indistinct details, making it difficult to interpret and visually distracting. The overall attractiveness of the image is distracting and impairs my ability to concentrate on the questions. (1 = Strongly disagree; 7 = Strongly agree)	0.775/0.773/0.747	0.662	0.809	0.585	Zhou et al. (2022)
Gender	1. Male 5. Female	-	-	-	-	-
Age	___________years	-	-	-	-	-
Education	1. High school or below 2. Junior college 3. Bachelor’s degree 4. Master’s degree 5. Doctoral degree	-	-	-	-	-
Occupation	1. Employee 2. Self-employed/Business owner 3. Student 4. Teacher 5. Other	-	-	-	-	-
Personal Income	1. Below ¥3000 2. ¥3001–¥5000 3. ¥5001–¥7000 4. ¥7001–¥9000 5. Above ¥9000	-	-	-	-	-

### Appendix H.4. Correlation Analysis

**Table A12:** Correlation Analysis in Study 3.

	V1	V2	V3	V4	V5	V6	V7	V8	V9	V10	V11
V1: Social Crowding	1										
V2: Shared Consumption	−0.037	1									
V3: Resource Mindset	−0.030	0.032	1								
V4: Price Difference	0.018	0.423 **	0.047	1							
V5: Emotional state (calm–anxious)	0.019	0.157 **	0.004	0.096	1						
V6: Image quality, clarity, and visual appeal	0.051	−0.056	0.050	−0.003	0.064	1					
V7: Gender	0.143 *	−0.130 *	−0.047	−0.073	−0.008	0.026	1				
V8: Age	0.058	−0.033	−0.046	−0.061	0.033	−0.038	0.069	1			
V9: Education	0.055	−0.095	−0.002	−0.080	0.079	0.090	0.040	−0.060	1		
V10: Occupation	−0.057	−0.013	0.021	0.077	0.030	−0.043	−0.053	0.043	−0.250 **	1	
V11: Personal Income	−0.065	0.090	0.046	0.058	0.056	−0.051	−0.063	−0.083	−0.118 *	0.016	1

*. The correlation is significant at the 0.05 level (two-tailed). **. The correlation is significant at the 0.01 level (two-tailed).

## Appendix I. Additional Study 1

Additional Study 1 extended the investigation of social crowding’s influence on shared consumption by examining whether social crowding fosters sharing-in, whereas non-social crowding encourages sharing-out. Using a controlled online scenario, interpersonal closeness was manipulated following Boothby et al. (2016) to simulate a sense of shared community. Participants’ willingness to share personal sunscreen served as the dependent measure, providing a precise test of social crowding effects in a virtual yet ecologically meaningful setting.

### Appendix I.1. Design and Participants

Additional Study 1 lasted 21 days and took place between 6 October and 4 November 2025. To expand the scope of the experiment, 300 participants were recruited via Credamo (credamo.com) from Southern China and compensated 1 RMB each. 28 participants who did not meet the experimental requirements were excluded from the analyses. Finally, a total of 272 Chinese participants (M*_Age_* = 32.50; SD = 8.03; 50.4% female; see Table A15 in Appendix J) were included in the analysis. Power analysis using G*Power 3.1 indicated a minimum of 179 participants for a medium effect size (*f* = 0.25) with 80% power, *α* = 0.05. The study employed a 2 (Social Crowding: crowded vs. uncrowded) × 2 (Shared Community: close friends vs. strangers) between-subjects design. The study was approved by the relevant ethics committee.

### Appendix I.2. Procedure

Social crowding was manipulated via photographic stimuli (see Appendix J). Participants were asked to vividly imagine themselves in the depicted setting, describing crowd density, interpersonal distance, and perceived personal space. The crowded condition depicted a densely populated beach (see Figure A9a in Appendix J); the uncrowded condition depicted a sparsely attended beach (see Figure A9b in Appendix J). Perceived crowding was assessed with two items: “How do you feel about the environment shown in the image?” and “To what extent is the environment in the image crowded?” (1 = Not at all crowded; 7 = Very crowded; α = 0.963, *CR* = 0.965, *AVE* = 0.932).

Shared community was manipulated through scenario prompts (see Appendix J.1 in Appendix J). Participants in the close friends condition read: “Please imagine you are at the beach with a family member or friend. You have your own bottle of sunscreen. When needed, would you be willing to share it with them?” In the strangers condition, the prompt replaced “family member or friend” with “someone you do not know.” Perceived closeness was measured using items adapted from Boothby et al. (2016), such as: “You feel close to the person(s) in the image,” “You trust them,” and “You feel understood by them” (1 = Strongly disagree; 7 = Strongly agree; α = 0.966, *CR* = 0.967, *AVE* = 0.908), adapted from Boothby et al. (2016).

Willingness to share was measured using a sunscreen-sharing scenario (see Figure A7). Willingness to share was measured with items adapted from Lteif et al. (2024), including: “You are willing to share this product with others,” “You think it is appropriate to share this product with others,” and “You are willing to continue sharing this product with others” (1 = Strongly disagree; 7 = Strongly agree; α = 0.956, *CR* = 0.958, *AVE* = 0.883). Control variables included image quality, clarity, and visual appeal (α = 0.633, *CR* = 0.835, *AVE* = 0.631; Zhou et al., 2022), familiarity with the product (α = 0.583, *CR* = 0.614, *AVE* = 0.448), and emotional state (negative-positive; α = 0.701, *CR* = 0.709, *AVE* = 0.551) to eliminate alternative explanations. Participants completed demographic questions at the end of the procedure.

### Appendix I.3. Results

#### Appendix I.3.1. Manipulation Checks

Social crowding: A 2 (Social Crowding: crowded vs. uncrowded) × 2 (Shared Community: close friends vs. strangers) ANOVA revealed a significant main effect of social crowding on perceived crowding, F (1, 268) = 73.85, *p* < 0.001, *η_p_*^2^ = 0.22. Neither the main effect of shared community, F (1, 268) = 2.53, *p* = 0.11, nor the interaction effect, F (1, 268) = 0.02, *p* = 0.89, was significant. These results confirm effective manipulation of social crowding.

Shared community: The same ANOVA on perceived closeness revealed a significant main effect of shared community, F (1, 268) = 74.57, *p* < 0.001, *η_p_*^2^ = 0.22. The main effect of social crowding was significant, F (1, 268) = 4.88, *p* < 0.05, *η_p_*^2^ = 0.02, but their interaction was nonsignificant, F (1, 268) = 1.95, *p* = 0.16, confirming the effectiveness of the shared community manipulation.

#### Appendix I.3.2. Correlation Analysis

Table A14 (see Appendix J) reports the bivariate correlations between social crowding, shared consumption, and the remaining study variables. The results indicate that willingness to share is significantly and positively associated with familiarity with the shared community (*r* = 0.472, *p* < 0.01) and familiarity with the product (*r* = 0.350, *p* < 0.01).

#### Appendix I.3.3. Willingness to Share

A 2 (Social Crowding: crowded vs. uncrowded) × 2 (Shared Community: close friends vs. strangers) ANOVA was conducted to examine the effects of social crowding and shared community on willingness to share. Significant main effects were found for both social crowding, F (1, 268) = 14.46, *p* < 0.001, *η_p_*^2^ = 0.05, and shared community, F (1, 268) = 282.98, *p* < 0.001, *η_p_*^2^ = 0.51. Importantly, the interaction effect was also significant, F (1, 268) = 104.65, *p* < 0.001, *η_p_*^2^ = 0.28.

Specifically, in the close friends condition, participants in the crowded setting reported significantly greater willingness to share (M*_Crowded_* = 6.25, SD = 0.44) than those in the uncrowded setting (M*_Uncrowded_* = 5.39, SD = 1.22), t (268) = 5.51, *p* < 0.001, 95% CI [0.55, 1.17], *d* = 0.94. Conversely, in the strangers condition, participants in the uncrowded condition reported significantly higher willingness to share (M*_Uncrowded_* = 4.50, SD = 1.33) than those in the crowded condition (M*_Crowded_* = 2.62, SD = 1.22), t (268) = −8.64, *p* < 0.001, 95% CI [−2.32, −1.45], *d* = −1.48. These findings support H1 (see Figure A8).

#### Appendix I.3.4. Analysis of Control Variable

Two-factor analysis of variance revealed no significant differences in emotional state (negative-positive; F (1, 270) = 1.66, *p* = 0.20), familiarity with the product (F (1, 270) = 3.11, *p* = 0.08), and image quality, clarity, and visual appeal (F (1, 270) = 1.66, *p* = 0.20).

### Appendix I.4. Discussion

By manipulating both environmental crowding and relational context, Additional Study 1 confirmed that social crowding increases willingness to share with close friends, whereas non-social crowding promotes sharing with strangers. The online scenario allowed control over potential confounds such as emotional state (negative—positive), familiarity with the product, and image quality, bolstering internal validity.

## Appendix J

### Appendix J.1. Manipulation Check Items for Additional Study 1

**Experimental situations (Group 1: Crowded × Close Friends)** *1. Please look at the picture, imagine that you are in the setting, and answer the following questions.* How do you feel about the environment shown in the image? To what extent is the environment in the image crowded? (1 = Not at all crowded, 7 = Very crowded) *2. Please imagine that you are at the beach with a family member or friend. You have a bottle of your own sunscreen. When it is needed, would you be willing to share it with them?* 1. You feel close to the person(s) in the image. 2. You trust them. 3. You feel understood by them. (1 = Strongly disagree, 7 = Strongly agree)
**Experimental situations (Group 2: Crowded × Strangers)** *1. Please look at the picture, imagine that you are in the setting, and answer the following questions.* How do you feel about the environment shown in the image? To what extent is the environment in the image crowded? (1 = Not at all crowded, 7 = Very crowded) *2. Please imagine that you are at the beach with someone you do not know. You have a bottle of your own sunscreen. When it is needed, would you be willing to share it with them?* 1. You feel close to the person(s) in the image. 2. You trust them. 3. You feel understood by them. (1 = Strongly disagree, 7 = Strongly agree)
**Experimental situations (Group 3: Uncrowded × Close Friends)** *1. Please look at the picture, imagine that you are in the setting, and answer the following questions.* How do you feel about the environment shown in the image? To what extent is the environment in the image crowded? (1 = Not at all crowded, 7 = Very crowded). *2. Please imagine that you are at the beach with a family member or friend. You have a bottle of your own sunscreen. When it is needed, would you be willing to share it with them?* 1. You feel close to the person(s) in the image. 2. You trust them. 3. You feel understood by them. (1 = Strongly disagree, 7 = Strongly agree)
**Experimental situations (Group 4: Uncrowded × Strangers)** *1. Please look at the picture, imagine that you are in the setting, and answer the following questions.* How do you feel about the environment shown in the image? To what extent is the environment in the image crowded? (1 = Not at all crowded, 7 = Very crowded). *2. Please imagine that you are at the beach with someone you do not know. You have a bottle of your own sunscreen. When it is needed, would you be willing to share it with them?* 1. You feel close to the person(s) in the image. 2. You trust them. 3. You feel understood by them. (1 = Strongly disagree, 7 = Strongly agree)

### Appendix J.3. Item Scale

**Table A13:** Measurement Items for Additional Study 1.

Variables	Measurement Items	Factor Loading	*α*	*CR*	*AVE*	References
Social Crowding (IV)	1. How do you feel about the environment shown in the image? 2. To what extent is the environment in the image crowded? (1 = Not at all crowded, 7 = Very crowded).	0.969/0.962	0.963	0.965	0.932	Maeng et al. (2013)
Shared Community	1. You feel close to the person(s) in the image. 2. You trust them. 3. You feel understood by them. (1 = Strongly disagree, 7 = Strongly agree).	0.948/0.962/ 0.948	0.966	0.967	0.908	Boothby et al. (2016)
Willingness to share (DV)	1. You are willing to share this product with others 2. You think it is appropriate to share this product with others 3. You are willing to continue sharing this product with others (1 = Strongly disagree, 7 = Strongly agree)	0.944/0.931/ 0.944	0.956	0.958	0.883	Lteif et al. (2024)
Emotional state	Please indicate the extent to which you experienced the following emotions in this environment: 1. I felt negative. 2. I felt positive. (1 = Very slightly or not at all; 7 = Extremely)	0.801/0.679	0.701	0.709	0.551	Watson et al. (1998)
Image quality, clarity, and visual appeal	1. The image colors fail to accurately convey topic-relevant information and lack visual appeal. 2. The image is unclear, lacks sufficient resolution, and contains indistinct details, making it difficult to interpret and visually distracting. 3. The graphic design elicits an emotional response. (1 = Strongly disagree; 7 = Strongly agree)	0.923/0.707/0.735	0.633	0.835	0.631	Zhou et al. (2022)
Familiarity with the product	1. I have previously seen this bottle of sunscreen. 2. I am very fond of this bottle of sunscreen. (1 = Strongly disagree; 7 = Strongly agree)	0.562/0.762	0.583	0.614	0.448	-
Gender	1. Male 5. Female	-	-	-	-	-
Age	___________years	-	-	-	-	-
Education	1. High school or below 2. Junior college 3. Bachelor’s degree 4. Master’s degree 5. Doctoral degree	-	-	-	-	-
Occupation	1. Employee 2. Self-employed/Business owner 3. Student 4. Teacher 5. Other	-	-	-	-	-
Personal Income	1. Below ¥3000 2. ¥3001–¥5000 3. ¥5001–¥7000 4. ¥7001–¥9000 5. Above ¥9000	-	-	-	-	-

### Appendix J.4. Correlation Analysis

**Table A14:** Correlation Analysis in Additional Study 1.

	V1	V2	V3	V4	V5	V6	V7	V8	V9	V10	V11
V1: Social Crowding	1										
V2: Willingness to share	−0.136 *	1									
V3: Shared Community	0.076	0.472 **	1								
V4: Emotional state (negative- positive)	0.079	−0.056	−0.030	1							
V5: Image quality, clarity, and visual appeal	0.164 **	−0.074	−0.101	0.124 *	1						
V6: Familiarity with the product	−0.072	0.350 **	0.223 **	0.004	−0.108	1					
V7: Gender	−0.013	0.036	−0.052	−0.075	0.000	−0.058	1				
V8: Age	0.013	0.110	0.068	−0.032	0.199 **	0.120 *	0.031	1			
V9: Education	0.112	0.005	0.133 *	0.044	−0.099	−0.060	0.031	−0.122 *	1		
V10: Occupation	0.140 *	−0.026	0.012	−0.119 *	0.053	0.045	−0.056	0.012	0.025	1	
V11: Personal Income	0.034	0.112	0.090	−0.021	0.046	0.164 **	−0.036	0.514 **	0.206**	−0.248 **	1

*. The correlation is significant at the 0.05 level (two-tailed). **. The correlation is significant at the 0.01 level (two-tailed).

### Appendix J.5. Descriptive Statistics for Additional Study 1

**Table A15:** Descriptive Statistics for Additional Study 1.

Additional Study 1					
N = 272, 50.4% Females, M*_Age_* = 32.50 Years, SD = 8.03					
		M (SD)		*df*	*p*
Variables		Crowded	Uncrowded		
Social Crowding		5.46 (1.21)	4.07 (0.12)	F (1, 268) = 73.85	0.001
Shared Community	Close Friends	5.50 (1.19)		F (1, 268) = 74.57	0.001
	Strangers	4.05 (1.59)			
Willingness to share	Close Friends	6.25 (0.44)	5.39 (1.22)	t (134) = 5.51	<0.001
	Strangers	2.62 (1.22)	4.50 (1.33)	t (134) = −8.64	<0.001
Emotional state (negative- positive)	Close Friends	3.63 (0.71)	3.59 (0.73)	t (134) = 0.30	=0.77
	Strangers	3.78 (0.86)	3.57 (0.80)	t (134) = 1.45	=0.15
Familiarity with the product	Close Friends	4.07 (1.28)	4.10 (1.12)	t (134) = −0.11	=0.92
	Strangers	3.32 (1.06)	3.82 (1.22)	t (134) = −2.51	=0.13
Image quality, clarity, and visual appeal	Close Friends	3.74 (0.89)	3.69 (0.94)	t (134) = 0.31	=0.76
	Strangers	4.01 (1.10)	3.76 (0.87)	t (134) = 1.47	=0.14
Gender	Close Friends	3.12 (2.01)	3.35 (1.99)	t (134) = 0.17	=0.87
	Strangers	3.18 (2.01)	3.41 (1.97)	t (134) = 0.68	=0.50
Age	Close Friends	33.09 (8.03)	33.04 (8.66)	t (134) = 0.03	=0.98
	Strangers	31.22 (7.26)	32.65 (8.15)	t (134) = 1.08	=0.28
Education	Close Friends	3.03 (0.60)	3.01 (0.47)	t (134) = 0.16	=0.87
	Strangers	3.06 (0.45)	2.97 (0.55)	t (134) = 1.03	=0.31
Familiarity with the product	Close Friends	4.07 (1.28)	4.10 (1.12)	t (134) = −0.11	=0.92
	Strangers	3.32 (1.06)	3.82 (1.22)	t (134) = −2.51	=0.13
Occupation	Close Friends	1.81 (1.27)	1.88 (1.39)	t (134) = 0.32	=0.75
	Strangers	1.81 (1.36)	1.37 (0.90)	t (134) = 2.23	=0.03
Personal Income	Close Friends	3.62 (1.52)	3.63 (1.47)	t (134) = 0.06	=0.95
	Strangers	3.47 (1.38)	3.59 (1.33)	t (134) = 0.51	=0.61

Notes: Statistics in parentheses are standard deviations.
